# Antibodies against a short region of PfRipr inhibit *Plasmodium falciparum* merozoite invasion and PfRipr interaction with Rh5 and SEMA7A

**DOI:** 10.1038/s41598-020-63611-6

**Published:** 2020-04-20

**Authors:** Hikaru Nagaoka, Bernard N. Kanoi, Edward H. Ntege, Masamitsu Aoki, Akihisa Fukushima, Takafumi Tsuboi, Eizo Takashima

**Affiliations:** 1 Division of Malaria Research, Proteo-Science Center, Ehime University, 3 Bunkyo-cho, Matsuyama, Japan; 2 Sumitomo Dainippon Pharma Co., Ltd, 3-1-98, Kasugadenaka, Konohanaku, Osaka 554-0022 Japan; 3 Present Address: Department of Plastic and Reconstructive Surgery, University of the Ryukyus, School of Medicine and Hospital, Okinawa, Japan

**Keywords:** Parasite biology, Protein vaccines

## Abstract

*Plasmodium falciparum* merozoite invasion into erythrocytes is an essential step of the blood-stage cycle, survival of parasites, and malaria pathogenesis. *P. falciparum* merozoite Rh5 interacting protein (PfRipr) forms a complex with Rh5 and CyRPA in sequential molecular events leading to erythrocyte invasion. Recently we described PfRipr as a conserved protein that induces strain-transcending growth inhibitory antibodies in *in vitro* assays. However, being a large and complex protein of 1086 amino acids (aa) with 87 cysteine residues, PfRipr is difficult to express in conventional expression systems towards vaccine development. In this study we sought to identify the most potent region of PfRipr that could be developed to overcome difficulties related to protein expression, as well as to elucidate the invasion inhibitory mechanism of anti-PfRipr antibodies. Using the wheat germ cell-free system, Ecto- PfRipr and truncates of approximately 200 aa were expressed as soluble proteins. We demonstrate that antibodies against PfRipr truncate 5 (PfRipr_5: C_720_-D_934_), a region within the PfRipr C-terminal EGF-like domains, potently inhibit merozoite invasion. Furthermore, the antibodies strongly block PfRipr/Rh5 interaction, as well as that between PfRipr and its erythrocyte-surface receptor, SEMA7A. Taken together, PfRipr_5 is a potential candidate for further development as a blood-stage malaria vaccine.

## Introduction

*Plasmodium falciparum* malaria remains a serious challenge to global health. In 2016, more than 3 billion people were reportedly at risk of infection, with an estimated 200 million cases and more than 400,000 deaths, primarily in young children living in sub-Saharan Africa^1^. Development of a malaria vaccine of high efficacy is considered a critical global agenda towards the achievement of malaria control and elimination. However, this progress has been greatly hampered by antigen polymorphism and low efficacy among target antigens^2,3^. Relatively conserved antigens that induce broadly cross-reactive antibodies and cell-mediated immune responses may provide long lasting and more efficacious protection^4–8^. It is also suggested that the next generation vaccines should incorporate multi-stage and/or multivalent targets aimed at inducing both humoral and cellular immunity^9^.

The process of merozoite invasion of erythrocytes, that marks the beginning of the blood stage cycle of *P. falciparum* infections, takes less than 2 min and is characterized by dynamic molecular and cellular events^10–12^. Upon egress from the infected erythrocyte the merozoite is exposed to low potassium levels which trigger intracellular calcium release. The release activates secretion of adhesins and invasins, localized in the micronemes, onto the parasite surface^13–15^. Following the initial recognition of the host erythrocyte the parasite orients itself so that its apical end is directly facing the target erythrocyte membrane. The rhoptries are subsequently triggered to release invasion ligands that interact with erythrocyte receptors^16,17^. Irreversible attachment of merozoites to erythrocytes then occurs through formation of an electron-dense nexus between the host and parasite membranes, termed a tight junction and composed primarily of PfAMA1 and RON complex proteins^15^. The nexus opens out into a ring-like moving junction, which envelops the merozoite, finally resealing behind it, such that the parasite is completely internalized within an intracellular parasitophorous vacuole^18^. Upon successful invasion echinocytosis occurs, most likely caused by entry of Ca^2+^ into the erythrocyte, resulting in shrinkage of the infected erythrocyte^10,11^. Because at the blood stage cycle the merozoites are exposed to various host immune responses, albeit for a short period between host cell egress and invasion, it is considered an ideal target for vaccine development^12,16^.

Investigations towards receptor-ligand interactions during merozoite invasion suggest that, in addition to merozoite surface proteins (MSPs), two protein ligand families play key roles prior to tight junction formation; namely, *P. falciparum* reticulocyte-binding protein homologs (PfRhs) and erythrocyte-binding like proteins (EBLs)^11,18,19^. The PfRhs include PfRh1, PfRh2a, PfRh2b, PfRh3, PfRh4, and Rh5. Rh5, the smallest member of the PfRhs, is localized within the merozoite rhoptries where it relocates to the moving junction during invasion^20^. Unlike genes encoding other PfRhs and EBLs, although functionally important, only the gene encoding Rh5 (PF3D7_0424100) is refractory to targeted gene deletion^21,22^. These reports suggest that the protein plays a crucial role in parasite survival, and this is supported functionally by confirmed binding to basigin on erythrocytes^23^. In addition, Rh5 forms a complex with *P. falciparum* Rh5-interacting protein (PfRipr)^24,25^ and cysteine-rich protective antigen (CyRPA)^5,26^. Rh5, CyRPA, and PfRipr are now considered to be promising blood-stage vaccine candidates^5–7,24,26,27^, since the PfRipr/CyRPA/Rh5 complex plays a central role in the sequential molecular events leading to merozoite invasion and the genes have limited sequence polymorphism in *P. falciparum*^5,27,28^.

Among Rh5, CyRPA, and PfRipr, the latter molecule is the least characterized because of its large size compounded by its cysteine-rich structure. PfRipr is a 126-kDa protein which localizes to the merozoite micronemes and contains ten epidermal growth factor-like (EGF-like) domains that are processed into two polypeptides. The processed 65-kDa PfRipr pair, which remains associated with Rh5 and CyRPA, is shed into the supernatant during invasion^24,25^. Although it is unclear how PfRipr interacts with CyRPA and Rh5, it has been demonstrated that the three proteins elicit invasion-inhibitory antibodies in experimental animals and naturally acquired antibodies in humans associate with protection from clinical malaria^5,7,24,26,27,29^. Noteworthy, when compared to Rh5 and CyRPA, antibodies against recombinant PfRipr have consistently shown higher *in vitro* growth inhibition assay (GIA) activity^5,7,24,26,27,29,30^. However, the mechanism by which anti-PfRipr antibodies induce such high GIA activity remains unknown.

Here, we investigated the mechanism of anti-PfRipr antibodies in inhibiting merozoite invasion through functional characterization of PfRipr. We utilized both wheat germ cell-free system (WGCFS)-expressed recombinant PfRipr protein and anti-PfRipr antibodies to assess GIA activity *in vitro*. Additionally, we applied WGCFS and AlphaScreen, a protein-protein based interaction screening system, and surface plasmon resonance (SPR) to systematically screen for and validate potential receptors of PfRipr on the surface of human erythrocytes. We demonstrated that PfRipr directly interacts with recombinant proteins of three erythrocyte surface proteins. Antibodies to a region within the PfRipr C-terminal EGF-like domains inhibited merozoite invasion and PfRipr association with Rh5 and SEMA7A. Taken together, the identified PfRipr region represents a promising candidate for further development as a blood-stage malaria vaccine.

## Results

### Antibodies against the C-terminal region of PfRipr from residues C_720_ to D_934_ exhibit growth inhibitory activity

**Figure 1:**
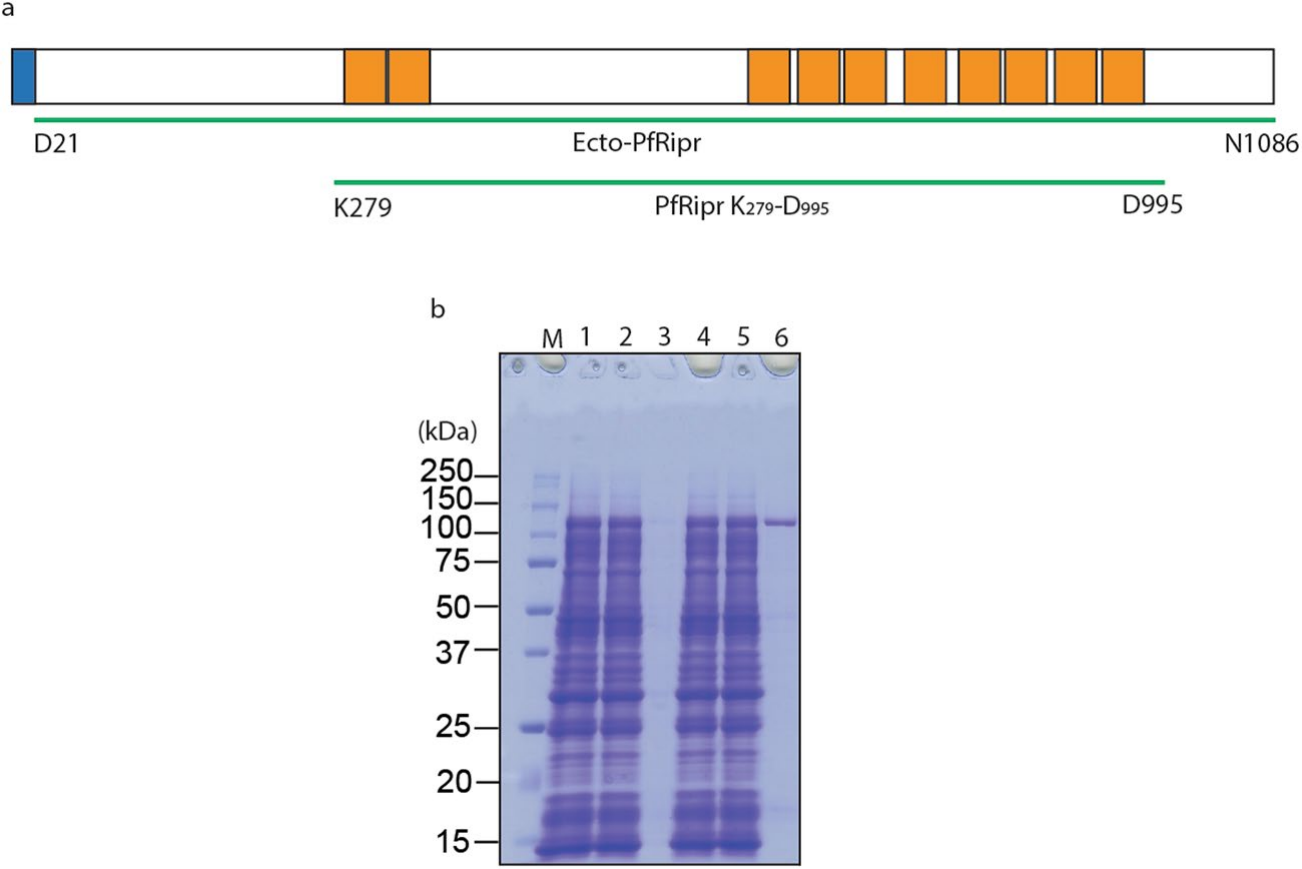
Primary structure and expression of PfRipr. (**a**) Design of PfRipr recombinant proteins. The PfRipr protein consists of 1086 aa with a calculated molecular mass of 125.9 kDa. The protein has a predicted signal peptide (SP; 1 to 20 aa) and ten EGF-like domains (orange). Recombinant Ecto-PfRipr; D_21_-N_1086_ (residues 21 to 1086), and PfRipr, K_279_-D_995_^30^ were expressed. (**b**) Expression of Ecto-PfRipr recombinant protein. Recombinant Ecto-PfRipr was expressed as C-terminal His-tagged protein using the wheat germ cell-free system (WGCFS), purified with Ni-affinity columns, resolved by 12.5% SDS-PAGE under reducing conditions, and stained with Coomassie brilliant blue (CBB). Lane 1 and 2: total translation mixture; Lane 3: purification pellet; Lanes 4, 5, and 6 represent supernatant, flow through, and elution fractions, respectively.

**Figure 2:**
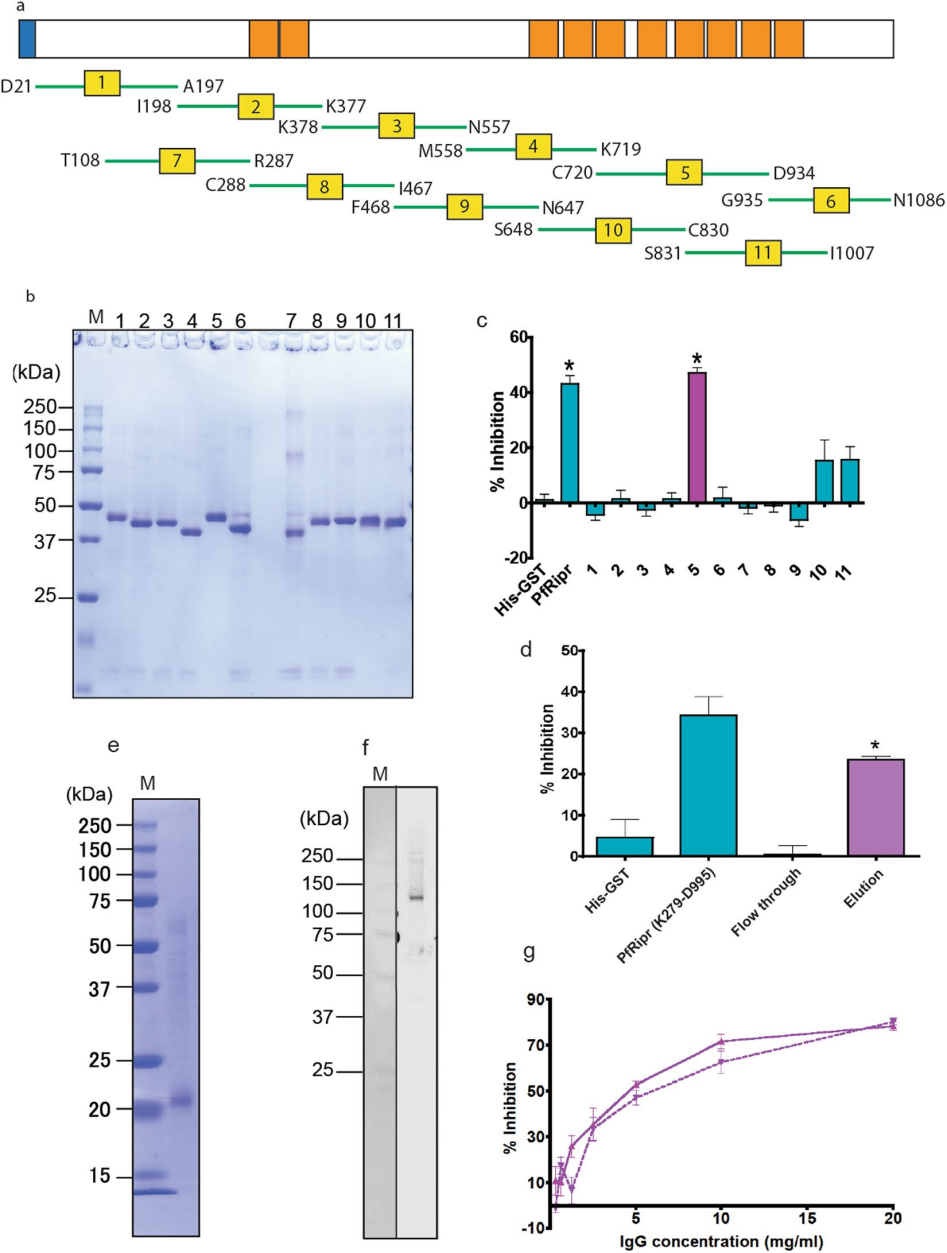
Assessment of truncated recombinant PfRipr for GIA. (**a**) Design of PfRipr truncates. Eleven overlapping fragments of PfRipr were designed as shown. (**b**) Expression of recombinant PfRipr truncates. PfRipr truncates were expressed by WGCFS as GST-fused proteins with a C-terminal His-tag, Ni^2+^ affinity purified, resolved by 12.5% SDS-PAGE under reducing conditions, and stained with CBB. Lanes 1–11 represent respective PfRipr truncates shown in Fig. 2a. M: molecular weight marker. (**c**) GIA activity of antibodies against PfRipr regions. The GIA activity of 10 mg/ml rat antibodies to each of the 11 PfRipr regions on *P. falciparum* 3D7 strain was compared. His-GST: negative control, anti-His-GST antibodies; PfRipr: anti-Ecto-PfRipr antibodies; 1–11: antibodies against each PfRipr truncate shown in Fig. 2b. Results are mean ± SEM of pooled data from six independent experiments. Error bar represents standard error of the mean. * Indicates statistically significant (Kruskal-Wallis test; P < 0.05) when compared against antibodies to His-GST. (**d**) GIA with purified PfRipr_5 antibodies and anti-PfRipr antibodies-depleted flow-through fraction. Eluate (0.72 mg/ml) and flow-through (10 mg/ml) fractions of rabbit anti-PfRipr K_279_-D_995_ antibodies purified with a recombinant PfRipr_5 immobilized column were compared in GIA. His-GST: rabbit anti-His-GST antibody as negative control; PfRipr K_279_-D_995_: anti-PfRipr K_279_-D_995_ antibodies, each at 10 mg/ml. Results are mean ± SEM of pooled data from three independent experiments. Error bar represents standard error of the mean. * Indicates statistically significant (Kruskal-Wallis test; P < 0.05) when compared against antibodies to His-GST. (**e**) Expression of PfRipr_5 truncate protein using baculovirus protein expression system (PfRipr_5-BPES). Purified recombinant PfRipr_5-BPES was resolved by SDS-PAGE under reducing conditions and stained with Coomassie brilliant blue (CBB). M: All Blue prestained protein molecular weight marker. (**f**) Reactivity of anti-PfRipr_5-BPES antibody on parasite PfRipr. Western blot analysis of PfRipr in trophozoite and schizont-rich parasite lysate using rabbit anti-PfRipr_5-BPES antibodies. M: molecular weight marker. (**g**) Dose dependent GIA activity of rabbit antibodies to PfRipr_5. GIA activities of anti-PfRipr_5 antibodies generated by BPES-expressed recombinant protein were measured at two-fold dilution; range 20 to 0.31 mg/ml. Solid red line, antibodies against PfRipr_5-BPES immunized at 0.1 mg/dose; dashed red line, antibodies against PfRipr_5-BPES immunized at 0.3 mg/dose.

To characterize PfRipr and to delineate the PfRipr region that is essential for GIA activity, as reported^30^, we produced Ecto-PfRipr as a His-tagged recombinant protein using WGCFS (Fig. 1a). Additionally, 11 approximately equal and overlapping PfRipr truncates were synthesized as N-terminal GST-fused and C-terminal His-tagged proteins (Fig. 2a). The expressed recombinant proteins were Ni^2+^ affinity purified as soluble proteins, resolved in 12.5% SDS-polyacrylamide gels, and stained with Coomassie brilliant blue R-250 (CBB). The Ecto-PfRipr protein was visualized as a single band of about 120 kDa (Fig. 1b, lane 6). The PfRipr truncate regions resolved at approximately similar molecular weights (Fig. 2b). Each purified recombinant PfRipr protein was used to immunize and raise antibodies in six rats. Sera from four rats which showed higher antibody titers were pooled and used in subsequent experiments.

The specificity of rat antibodies to detect native PfRipr was evaluated by Western blot using *P. falciparum* schizont-rich parasite lysates. Antibodies against the Ecto-PfRipr (Fig. 1b) and each of the 11 PfRipr regions (Fig. 2a) immunoprecipitated native PfRipr, which could be detected as a double band of approximately 70 and 120 kDa (Supplemental Fig. S1).

To evaluate whether rat anti-PfRipr antibodies could block parasite invasion in GIA, the antibodies were tested for inhibition of parasite growth over one cycle of replication as determined by flow cytometry. At a final concentration of 10 mg/ml (total IgG), antibodies to PfRipr C_720_-D_934_ (PfRipr_5) showed the highest GIA activity to the 3D7 strain by 46 ± 3% (mean ± SE); significantly higher than the negative control, anti-His-GST antibodies (P < 0.05; Fig. 2c). To further validate that antibodies against PfRipr_5 are responsible for the observed GIA activity, anti-PfRipr_5 antibodies were purified from rabbit anti-PfRipr K_279_ -D_995_ (Fig. 1a)^30^ using recombinant PfRipr_5 immobilized on a HiTrap NHS-activated HP column (GE Healthcare). PfRipr_5 specific antibodies at 0.7 mg/ml induced a GIA activity of up to 24 ± 1% (mean ± SE) (Fig. 2d), albeit lower than that reported for leading blood stage vaccine candidates; specifically, Rh5 has an inhibition IC50 of 9 µg/ml^31^ while CyRPA has an IC50 of 0.38 mg/ml of total IgG^32^. The anti-PfRipr_5 antibody-depleted flow-through fraction did not show detectable GIA activity, indicating that PfRipr_5 contains all epitopes of invasion blocking antibodies against PfRipr K_279_ -D_995_ antigen.

PfRipr_5 is a relatively small protein with the potential of being an easily produced vaccine, and thus was synthesized as a soluble protein using a baculovirus protein expression system (BPES) amenable for downstream vaccine development. Expression was confirmed by SDS-PAGE (Fig. 2e) and the recombinant protein was used to raise antibodies in rabbit. Antibody specificity was confirmed by recognition of native PfRipr at approximately 120 kDa in Western blot analysis using a schizont-rich parasite lysate (Fig. 2f). To evaluate the GIA activity of antibodies to BPES PfRipr_5, we performed GIA at two-fold serial dilutions ranging from 20 to 0.31 mg/ml. We observed a dose dependent inhibition (Fig. 2g). Indeed, at only 5 mg/ml we observed an inhibitory activity of up to 53 ± 1% and 47 ± 3% (mean ± SE) for 0.1 mg and 0.3 mg immunization doses, respectively (Fig. 2g). Put together, these results confirmed the GIA activity of anti-PfRipr antibodies and identified that PfRipr_5, spanning amino acids C_720_-D_934_, is the key target of the GIA activity inducing antibodies.

### PfRipr interacts directly with both Rh5 and CyRPA

**Figure 3:**
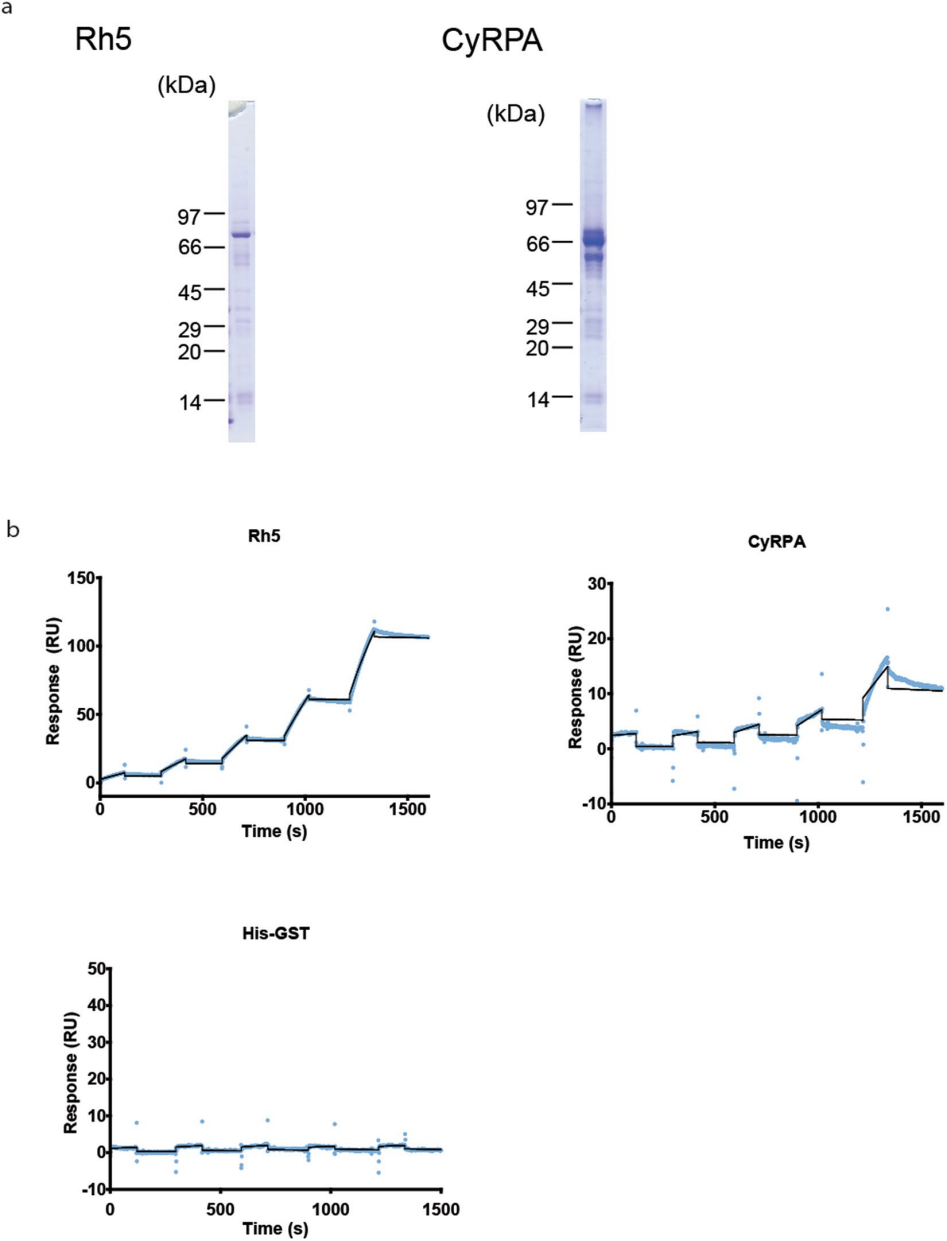
Interaction of PfRipr with Rh5 and CyRPA. (**a**) Recombinant Rh5 and CyRPA protein expression. Recombinant Rh5 and CyRPA proteins were expressed with WGCFS as GST-fused proteins with C-terminal His-tags. The proteins were resolved by 12.5% SDS-PAGE under reducing conditions and stained with CBB. Full-length SDS-PAGE images are presented in Supplementary Fig. S2. (**b**) SPR single-cycle kinetic analysis sensorgrams. Recombinant PfRipr immobilized on a sensor CM5 chip was used as the ligand while either Rh5, CyRPA, or His-GST was used as analyte. Blue dots represent the real data-generated sensorgram while the black curve indicates line of fit used to calculate kinetics parameters. All assays were performed at increasing protein concentrations of 3.13, 6.25, 12.5, 25, and 50 nM at a contact time of 120 s and dissociation time of 180 s. The last dissociation time was extended to 3000 s to accurately determine kinetic parameters (Fig. S3).

Based on several studies^5,26,33^, we sought to understand the nature of the complex formed between PfRipr, Rh5, and CyRPA, and if this interaction was direct or indirect. To demonstrate and quantify the interaction between PfRipr and Rh5, as well as with CyRPA, surface plasmon resonance (SPR) analysis was performed. Recombinant ecto regions of both Rh5 and CyRPA were expressed as GST-fusion proteins using WGCFS and affinity purified by virtue of the GST tag. The sizes of affinity purified recombinant Rh5 and CyRPA were as estimated (Figs. 3a, S2). However, contrary to a recent study^34^, we observed that PfRipr directly interacts with Rh5 at a *K*_D_ value of 4.8 × 10^−10^ M (Fig. 3b, Table 1, Fig. S3a). Comparably, PfRipr and CyRPA exhibited a *K*_D_ value of 6.9 × 10^−8^ M (Fig. 3b, Table 1, Fig. S3a). Thus, we demonstrated for the first time that PfRipr interacts directly with both Rh5 and CyRPA.

**Table 1:** Kinetic constants derived from SPR sensorgrams.

	*k*_a_ (1/Ms)	*k*_d_ (1/s)	*K*_D_ (M)	R_max_(RU)	${\boldsymbol{\chi }}$^2^ (RU^2^)
Ripr/Rh5	6.1 × 10^4^	2.9 × 10^−5^	4.8 × 10^−10^	211.6	0.996
Ripr/CyRPA	2.4 × 10^3^	1.6 × 10^−4^	6.9 × 10^−8^	428.4	0.649
Ripr/AMIGO2	1.2 × 10^5^	2.0 × 10^−5^	1.6 × 10^−10^	349.4	1.380
Ripr/SEMA7A	4.4 × 10^4^	4.1 × 10^−5^	9.4 × 10^−10^	609.4	3.860
Ripr/NPTN	2.7 × 10^3^	6.5 × 10^−5^	2.4 × 10^−8^	630.3	0.629
Ripr/HisGST	N/A	N/A	N/A	N/A	N/A
Ripr/CD47	N/A	N/A	N/A	N/A	N/A

### PfRipr binds to human erythrocytes

**Figure 4:**
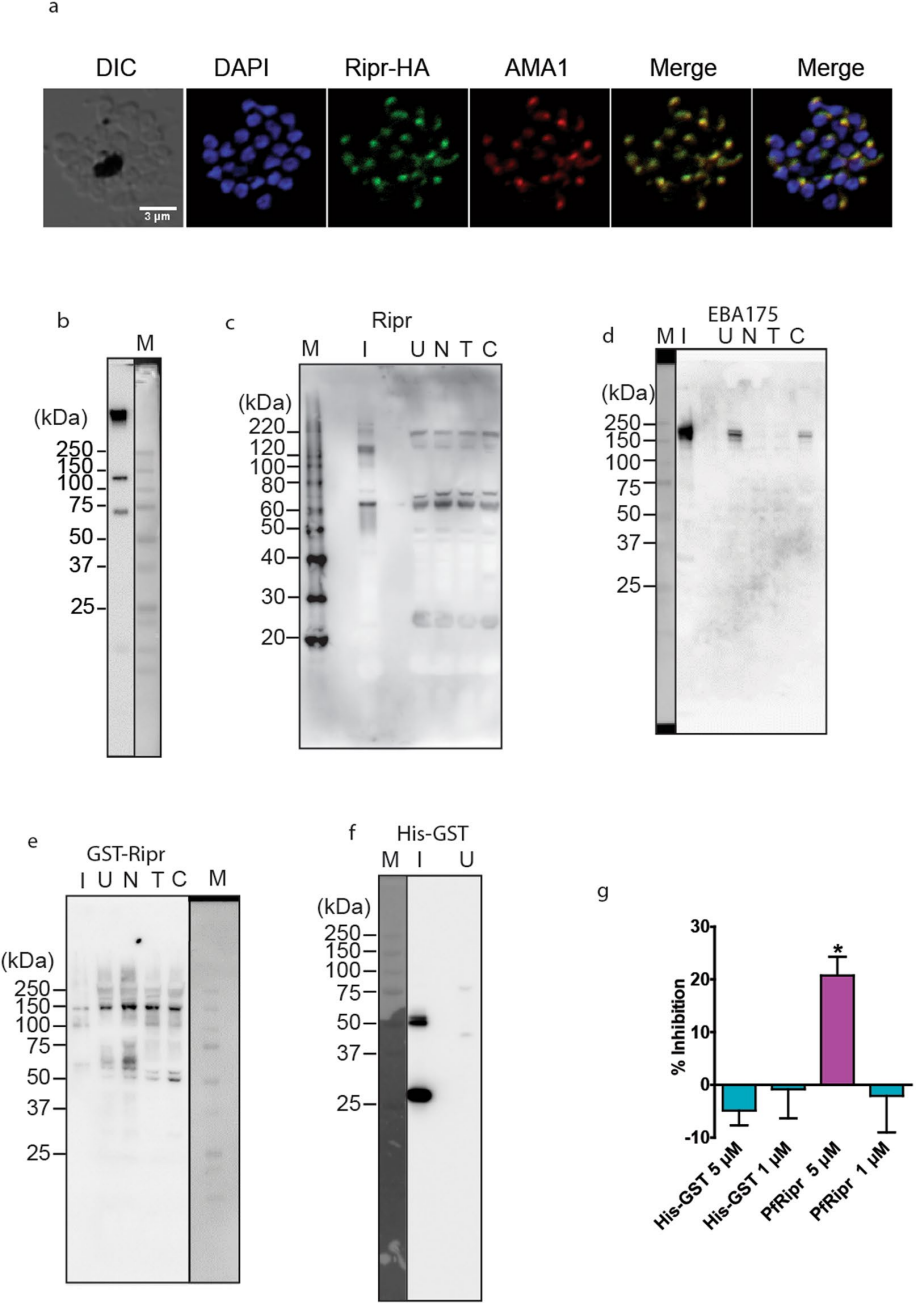
Erythrocyte binding assay for PfRipr. (**a**) PfRipr-HA localizati on. Paraformaldehyde-fixed mature schizonts from PfRipr-HA transfected *P. falciparum* were probed with rabbit anti-HA antibody (Ripr-HA: green) and co-stained with mouse antibodies to AMA1, a microneme marker (AMA1: red). The parasite nuclei were stained with DAPI (DAPI: blue). DIC; differential interference contrast. Scale bar = 3 µm. (**b)** Detection of PfRipr-HA in culture supernatant of PfRipr-HA transfected parasites. Culture supernatant was examined by Western blotting under reducing conditions with rabbit anti-HA antibodies. M: molecular weight marker. (**c,d**) Erythrocyte binding activity of PfRipr protein probed with anti-HA antibody (**c**) and EBA175 protein probed with anti-EBA175 antibody (**d**) obtained from parasite culture supernatant. I, parasite culture supernatant; U, untreated erythrocytes; N, neuraminidase treated erythrocytes; T, trypsin treated erythrocytes; C, chymotrypsin treated erythrocytes. M: molecular weight marker. (**e,f**) Erythrocyte binding activity of recombinant (**e**) PfRipr protein and (**f**) His-GST. I, recombinant protein; U, untreated erythrocytes; N, neuraminidase treated erythrocytes; T, trypsin treated erythrocytes; C, chymotrypsin treated erythrocytes. M: molecular weight marker. (**g**) GIA activity of recombinant proteins. Purified recombinant GST-fused PfRipr and *in vitro* cultured *P. falciparum* 3D7 strain. His-GST protein was used as a negative control. Error bar represents standard error of the mean. * Indicates statistically significant (Kruskal-Wallis test; P < 0.05) when compared to His-GST protein.

To understand the mechanism of invasion inhibition by anti-PfRipr antibodies, we sought to determine whether PfRipr binds directly to the surface of host erythrocytes. First, we generated transgenic parasite lines expressing full-length PfRipr (M_1_-N_1086_) with a C-terminal 3 × HA tag (PfRipr-HA). *In vivo* PfRipr-HA expression was confirmed by immunofluorescence assay (IFA), showing co-localization with AMA1 in the micronemes suggesting native PfRipr localization (Fig. 4a). We further observed that anti HA-tag could immunoprecipitate basigin and Rh5 (Fig. S4a). Since studies have indicated that culture supernatants can be a source of parasite proteins that bind host erythrocytes^35^, we used culture supernatants of the transgenic parasites to further confirm successful expression and release of PfRipr-HA into the culture supernatant (Fig. 4b). Consistent with the reported size of native PfRipr^5,24^, signals at approximately 70 and 120 kDa were observed (Fig. 4b). Although an earlier study had suggested that PfRipr is processed, and that the 60–70 kDa signal represents a processed C-terminal fragment, a recent study demonstrated that antibodies against the N-terminal and C-terminal EGF like domains of PfRipr detected the two signals, suggesting that the signals are derived from full-length PfRipr^5,24^ and the 70-kDa signal might be due to multiple disulfide bonds in PfRipr despite reducing conditions. The ratio of the 2 signals varied in each preparation of the SDS-PAGE sample even from same culture supernatant or parasite lysate (data not shown).

Second, we performed erythrocyte binding assays (EBA) to determine if PfRipr-HA could bind to erythrocytes. Neuraminidase treatment selectively removes sialic acid residues while trypsin and chymotrypsin treatments differentially cleave the peptide backbones of erythrocyte surface proteins^36–38^. Using culture supernatants, we observed that native PfRipr-HA binds erythrocytes in a chymotrypsin, neuraminidase, and trypsin resistant manner (Fig. 4c) indicating that binding is independent of enzyme-sensitive sites on the erythrocyte surface. As a control, binding of EBA175 to erythrocytes was sensitive to neuraminidase and trypsin treatment but was unaffected by chymotrypsin treatment (Fig. 4d).

To clarify if the observed interaction was a result of direct binding of PfRipr-HA to the erythrocyte surface, we examined the erythrocyte binding ability of recombinant Ecto-PfRipr at a final concentration of 35 nM. The results shown in Fig. 4e indicated that N-terminal GST-fused recombinant Ecto-PfRipr bound erythrocytes with similar enzyme treatment profiles as native PfRipr-HA (Fig. 4c), thus validating that native PfRipr may directly bind to erythrocytes. Recombinant His-GST was used as a negative control (Fig. 4f).

To assess the physiological role of the erythrocyte surface binding of PfRipr, we determined the GIA activity of N-terminal GST-fused recombinant PfRipr protein. The recombinant PfRipr significantly inhibited parasite growth by 21 ± 4% (mean ± SE) at 5 µM final protein concentration, which was significantly higher than that of His-GST, a negative control at the same concentration (P < 0.05; Fig. 4g). These findings (Fig. 4g) suggested at least two possibilities: (1) the recombinant Ecto-PfRipr could inhibit PfRipr interaction with parasite proteins, Rh5 and CyRPA, and (2) the recombinant Ecto-PfRipr inhibits interaction between parasite PfRipr with human erythrocyte surface receptor(s) during merozoite invasion. We sought to validate these postulations in subsequent assays.

**Figure 5:**
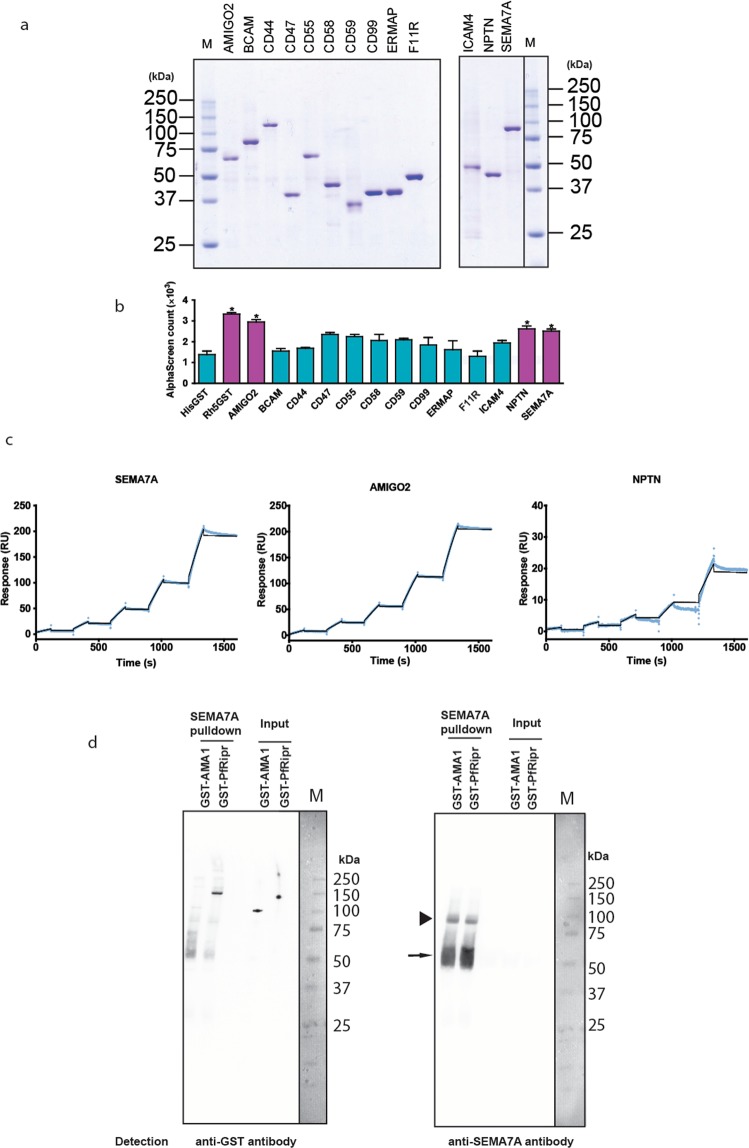
Identification of PfRipr binding receptor on the erythrocyte surface. (**a**) Thirteen major erythrocyte surface proteins were expressed with WGCFS as GST-fused proteins with C-terminal His-tags. Recombinant proteins purified using Ni-affinity columns were resolved by 12.5% SDS-PAGE under reducing conditions and CBB stained. Protein names are indicated above each lane. M: molecular weight marker. (**b**) AlphaScreen reactivity profile of recombinant PfRipr to 13 GST-fused recombinant proteins. Each bar represents the average AlphaScreen counts in quintuplicate with error bars representing SE of the mean. * Indicates statistically significant (Kruskal-Wallis test; P < 0.05) when compared to His-GST protein. (**c**) SPR single-cycle kinetic analysis sensorgrams. Recombinant PfRipr was immobilized on a CM5 chip and used as the ligand while either SEMA7A, AMIGO2, or NPTN was used as analyte. Blue dots represent the actual data-generated sensorgram while the black curve indicates the line of fit used to calculate kinetics parameters. All assays were performed at increasing protein concentrations of 3.13, 6.25, 12.5, 25, and 50 nM with a contact time of 120 s, and dissociation time of 180 s. The last dissociation time was extended to 3000 s to accurately determine kinetic parameters (Fig. S3). (**d**) Western blot analysis: erythrocytes ghosts were first mixed with recombinant GST-PfRipr, and SEMA7A was subsequently immunoprecipitated with rabbit anti-SEMA7A polyclonal antibodies. A parallel control experiment conducted with GST-AMA1 was included. SEMA7A pulldown; and immunoprecipitated sample, input. The samples were derived from erythrocyte ghosts mixed with recombinant GST-PfRipr or GST-AMA1. The membrane was probed with mouse anti-GST antibodies (left panel) and rabbit anti-SEMA7A antibodies (right panel) with membrane stripping, washing, and blocking in between. Due to the low amount of SEMA7A in the input lanes, no band was observed (right panel). Arrowhead; SEMA7A, arrow; heavy chain.

To identify potential PfRipr receptors on the host erythrocyte surface we used a systematic screening approach by first compiling a library of 13 abundantly expressed erythrocyte surface proteins^39^ and subsequently synthesizing them as N-terminal GST and C-terminal His-tagged recombinant proteins using WGCFS. Expression as soluble proteins was confirmed by SDS-PAGE (Fig. 5a). We then applied the AlphaScreen protein-protein interaction assay^40^ to screen for potential receptors binding PfRipr. Interaction between PfRipr and Rh5, which is known to exist, was used to demonstrate positive interaction while PfRipr interaction with His-GST was used as a negative control. Three erythrocyte surface proteins had significantly higher signals compared to the negative control (Kruskal-Wallis test; P < 0.05) (Fig. 5b), suggesting interaction with PfRipr; namely, adhesion molecule with IgG-like domain 2 (AMIGO2, also referred to as ALI1; DEGA), semaphorin-7A (SEMA7A; CD108), and neuroplastin (NPTN).

To validate the specificity of the interaction between PfRipr and erythrocyte surface protein we systematically analyzed the strength of interaction between the three erythrocyte surface proteins and PfRipr using surface plasmon resonance (SPR). We established that recombinant PfRipr (Fig. 1b, lane 6) directly interacts with recombinant AMIGO2, SEMA7A, and NPTN with equilibrium binding constant (*K*_D_) values of 1.6 × 10^−10^ M, 9.4 × 10^−10^ M, and 2.4 × 10^−8^ M, respectively (Fig. 5c, Table 1, Fig. S3b). SPR with CD47, which ranked highest among proteins with non-significant values in AlphaScreen (Fig. 5b), did not show any interaction with PfRipr (Fig. S3c). In summary, AMIGO2, SEMA7A, and NPTN represent putative receptors of PfRipr on the surface of host erythrocytes.

Using the recombinant Ecto-PfRipr protein and erythrocyte ghosts, immunoprecipitation experiments were conducted to confirm the above interactions. GST-fused Ecto-PfRipr and GST-fused AMA1 (negative control) were incubated with erythrocyte ghosts, then SEMA7A was pulled down with anti-SEMA7A antibodies. Anti-SEMA7A antibody successfully immunoprecipitated recombinant GST-fused Ecto-PfRipr (Fig. 5d, left panel) as well as native SEMA7A (Fig. 5d, right panel and Fig. S4b). The data suggests that recombinant PfRipr can bind native SEMA7A. This data is consistent with the SPR analysis. However, even though studies have suggested that AMIGO2 and NPTN proteins exist on the surface of erythrocytes^39^, multiple efforts were unsuccessful to detect the two proteins by Western blot, or by immunoprecipitation with PfRipr. We therefore focused only on characterization of PfRipr/SEMA7A interactions in further determinations of the potential mode of action of antibodies against PfRipr_5.

### Antibodies to PfRipr_5 block invasion by inhibiting interaction between PfRipr/Rh5 and PfRipr/SEMA7A

**Figure 6:**
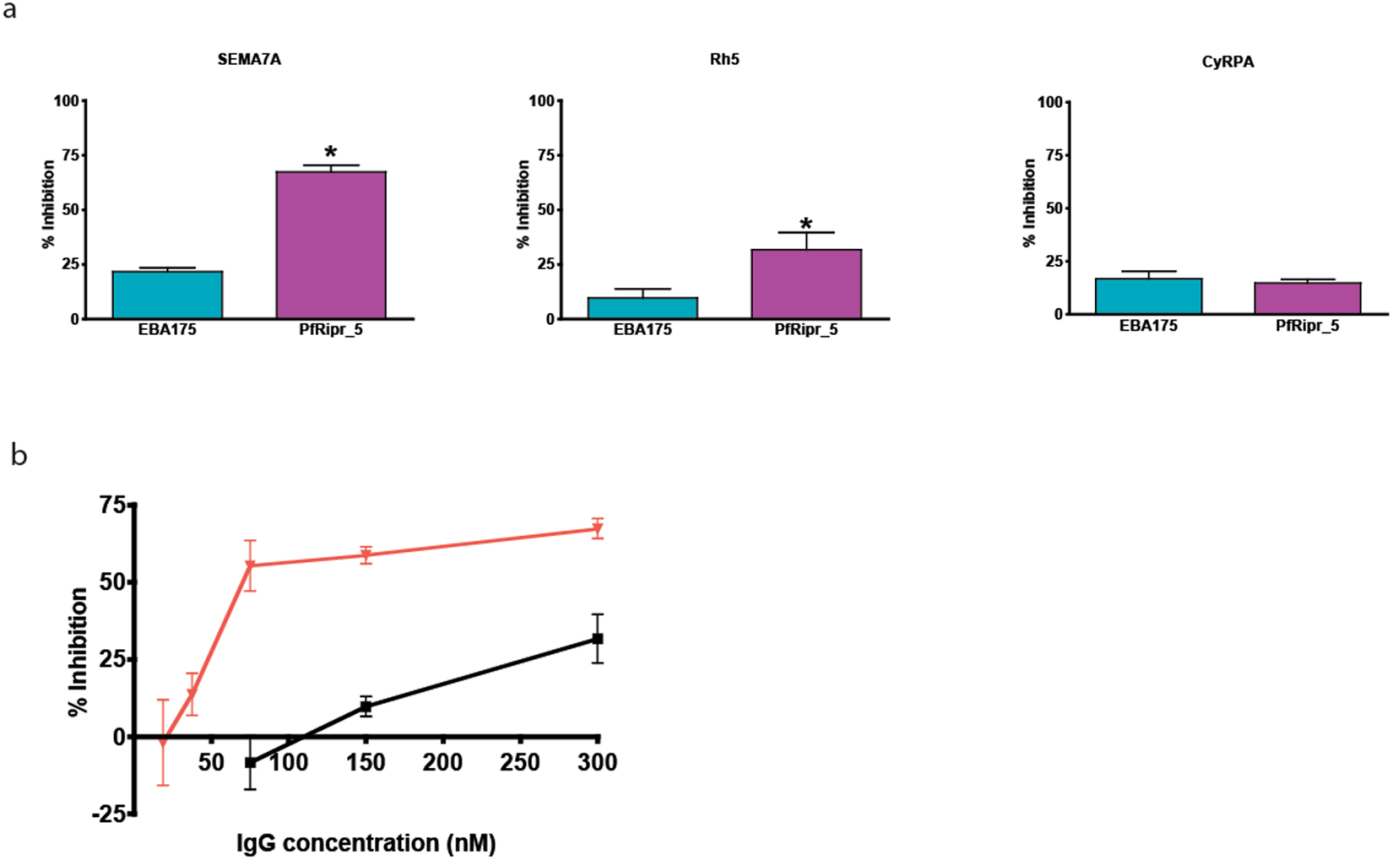
Inhibition of PfRipr binding on the erythrocyte receptor SEMA7A by anti-PfRipr_5 antibody. (**a**) Binding inhibition with anti-PfRipr_5 antibody. Effect of anti-PfRipr_5 antibodies (300 nM) to inhibit protein-protein interaction between 300 nM Ecto-PfRipr as analyte, and either SEMA7A, Rh5, or CyRPA as ligand by SPR. The sensor chip used was the same as in Fig. 3b. Each bar represents the average inhibition of three independent experiments. Error bars represent SE of the mean. * Indicates statistically significant by Student’s t test, P < 0.05. (**b**) Dose dependent binding-inhibitory activity of anti-PfRipr_5 antibody on PfRipr with SEMA7A and Rh5. Effects of antibodies to PfRipr_5 at a decreasing final concentration of 300 (presented in **a)** above), 150, 75.0, 37.5 and 18.8 nM were tested to inhibit protein-protein interaction between 300 nM PfRipr (analyte) and either Rh5 or SEMA7A by SPR. Red line, PfRipr and SEMA7A; black line, PfRipr and Rh5.

Having observed that PfRipr interacts directly with Rh5, CyRPA, and human erythrocyte protein SEMA7A, we sought to determine the mode of action of antibodies to PfRipr_5. Specifically, using SPR we assessed whether antibodies against PfRipr_5 could inhibit protein-protein interactions between PfRipr and either Rh5, CyRPA, or SEMA7A. Individual recombinant GST-fused proteins were used as ligands captured on SPR sensor chips with a GST-capture kit, followed by addition of analyte consisting of recombinant Ecto-PfRipr protein and anti-PfRipr_5 antibodies. As shown in Fig. 6a, anti-PfRipr_5 antibodies significantly inhibited interaction between both PfRipr/Rh5 and PfRipr/SEMA7A by 25 ± 5% and 58 ± 3% (mean ± SE; Student’s t-test P < 0.05), respectively. Anti-EBA175 (III-V) antibodies used as a negative control did not show significant inhibition. Antibodies against PfRipr_5 had no observed effect on PfRipr/CyRPA interaction (Fig. 6a). Additionally, dose dependence assays revealed that antibodies to PfRipr_5, at decreasing concentrations of 300, 150, 75, 37.5 and 18.8 nM, exerted an inhibitory effect on both PfRipr/Rh5 and PfRipr/SEMA7A interactions that decreased in a dose dependent manner (Fig. 6b). Taken together, the data presented here suggest that antibodies to PfRipr_5 block invasion by potentially inhibiting PfRipr/Rh5 interaction, as well as that between PfRipr and its novel erythrocyte-surface receptor SEMA7A. PfRipr_5 therefore offers an ideal target for further evaluation as a blood-stage malaria vaccine.

## Discussion

Invasion of erythrocytes by *P. falciparum* merozoites is a fundamental step in malaria pathogenesis and therefore a primary target for vaccine development^16,41^. Understanding biochemical interactions is key to identifying the role of proteins involved in the invasion process. One protein of particular interest is PfRipr, a leading subunit vaccine candidate and a vital protein in erythrocyte invasion^5,25,30,42^. We recently determined that PfRipr is highly conserved among African isolates, with the region spanning C_720_-D_934_ (PfRipr_5) having only a single amino acid substitution at E_829_Q^30^. The PfRipr/CyRPA/Rh5-basigin complex is suggested to play a central role in the sequential molecular events leading to merozoite invasion^25^. We and others have further demonstrated that specific antibodies raised against recombinant PfRipr exhibit strain-transcending inhibition of *P. falciparum in vitro* growth^25,30^. However, it is not understood how Rh5 and CyRPA interact with PfRipr, and how anti-PfRipr antibodies consistently achieve high GIA activity *in vitro*. Here, we investigated the mechanism of anti-PfRipr antibodies in inhibiting merozoite invasion. PfRipr, being a 126-kDa protein with 87 cysteine residues, is difficult to express as full-length protein in conventional expression systems. Therefore, we utilized the eukaryotic WGCFS to express PfRipr, which we subsequently subjected to systematic biochemical analyses. We demonstrated that antibodies to a short region of PfRipr (PfRipr_5: C_720_ - D_934_) in the C-terminal spanning five EGF-like domains (fifth to ninth EGF-like domains) inhibited merozoite invasion, as well as blocked interaction between PfRipr and its erythrocyte receptors. The GIA potency observed was lower than in a recent study^43^, an aspect that should be given careful consideration in future studies. Nevertheless, PfRipr_5 is a relatively small region that deserves further evaluation for its candidacy as a blood-stage vaccine antigen.

Previous studies indicated that PfRipr localizes to merozoite micronemes and is released onto the parasite surface during invasion, forming a tripartite complex with Rh5 and CyRPA^25^. The complex is then anchored to the surface of erythrocyte via basigin^5,24,44^. However, the data presented here is somewhat contradictory to those studies. Specifically, using the AVEXIS assay Galaway *et al*. did not observe direct interaction between PfRipr/Rh5^34^. Similarly, the Rh5/CyRPA/PfRipr complex structural analysis did not directly show PfRipr/Rh5 binding^45^. Nonetheless, they did observe that, after binding to basigin, the Rh5/CyRPA/PfRipr complex disassembled to generate an erythrocyte membrane associated Rh5, and PfRipr which migrated as a single band of high molecular weight (about 700 kDa), suggesting that they were in the same complex as oligomers. Based on the latter observation, our report on a PfRipr/Rh5 direct interaction may offer insight to the situational path of the Rh5/CyRPA/PfRipr complex after binding to basigin. Indeed, our EBA studies with episomally expressed PfRipr fused with an HA tag (Fig. 4c) is consistent with previous results showing that PfRipr was present within the erythrocyte membrane pellet^45^. We further demonstrated that GST-PfRipr binds to the erythrocyte surface and is resistant to chymotrypsin, trypsin, and neuraminidase treatment similar to PfRipr-HA. In this non-traditional EBA, we incubated recombinant GST-PfRipr with erythrocytes, washed, and then hemolyzed the cell with tetanolysin. The erythrocyte ghost was further washed with buffer, mixed with sample buffer and applied onto an SDS-PAGE gel. Thus, using AlphaScreen, we probed for interactions between biotinylated PfRipr ectodomain as bait and GST-fused erythrocyte surface receptors (Fig. 5a,b). Subsequently, analyses by SPR confirmed that PfRipr directly interacts with AMIGO2, SEMA7A, and NPTN (Fig. 5c). In addition, anti-SEMA7A antibody immunoprecipitated recombinant GST-PfRipr suggesting that recombinant PfRipr could bind native SEMA7A (Fig. 5d). Taken together, these findings suggest that PfRipr can function as a parasite ligand. These data need to be further validated since it could mean that (i) the native PfRipr indeed binds to erythrocyte SEMA7A, or (ii) recombinant GST-Ripr protein is structurally dissimilar from native PfRipr, perhaps due to lacking a parasite specific post-translational modification, giving it a different erythrocyte surface binding phenotype.

Attempts to genetically disrupt PfRipr have been unsuccessful, suggesting that it is essential for blood-stage parasite growth^24^. In addition, using *P. falciparum* lines conditionally expressing PfRipr, Volz *et al*. demonstrated that loss of PfRipr function blocks growth due to the inability of merozoites to invade erythrocytes^25^. Consistent with this, anti-PfRipr antibodies have been known to induce high GIA activity^30^. The key question is therefore, what is the mechanism of anti-PfRipr antibodies on parasite growth inhibition? In our study, anti-PfRipr polyclonal antibodies blocked the interaction between PfRipr and Rh5, as well as between PfRipr and a host receptor SEMA7A. It was therefore postulated that the antibody blockage of PfRipr binding to its multiple partners could explain the observed growth inhibition activity. However, we were unable to show a correlation between interaction inhibition in SPR and *in vitro* parasite GIA. Thus, in future studies we would like to address the mode of inhibition with monoclonal antibodies, as well as validate the *in vitro* SPR findings by an *in vivo* protein-protein interaction detection system.

SEMA7A, the John-Milton-Hagen blood group antigen, was recently reported as the receptor for *P. falciparum* merozoite-specific TRAP homolog, MTRAP^46^. SEMA7A is a GPI-anchored membrane bound protein expressed on several tissues with diverse functions; however, the functional role of the interaction remains unclear since antibodies to either MTRAP or SEMA7A did not cause inhibition in *in vitro* parasite invasion assays^46^. Similarly, our attempts to inhibit merozoite invasion with anti-SEMA7A antibodies showed no GIA activity, possibly due to insufficient antibody molecules which target SEMA7A surfaces important for PfRipr/SEMA7A interactions (Fig. S4c). Another possibility is that the inhibition caused by the anti-PfRipr_5 antibodies may not have been due to inhibition of PfRipr-SEMA7A interaction, but by another unknown mechanism such as anti-PfRipr antibodies inhibiting PfRipr/Rh5 interaction^43^. Our analysis showed that recombinant PfRipr/SEMA7A interacts at a nM level *K*_D_ (Fig. 5c); however, we could not immunoprecipitate a native PfRipr/Rh5/SEMA7A complex. This could suggest that the interaction is a “strong transient complex” and a trigger would be needed for its dissociation^47^. Indeed, anti-PfRipr_5 antibodies inhibit both PfRipr/Rh5 and PfRipr/SEMA7A interactions (Fig. 6a,b), suggesting that these PfRipr interacting regions are close. Recently, Healer *et al*. reported that neutralizing anti-PfRipr monoclonal antibody prevents merozoite invasion by not blocking Rh5/CyRPA/PfRipr complex formation but by another mechanism^43^. Although our results may support an alternative mechanism of blocking ligand-receptor interaction, this model needs confirmation. Still, the *K*_D_ values determined by SPR with recombinant PfRipr and SAMA7A proteins were low and suggested a high affinity interaction; however, this is generally not the case for natural ligand-receptor interactions which have low affinity adhesion^48^. To understand the PfRipr/SEMA7A interaction in detail, we may need further analysis for *K*_D_ values using other methods, for example radioligand binding assays.

In conclusion, we show that a small region of PfRipr (PfRipr_5: C_720_ - D_934_) located within the C-terminal EGF-like domains elicits antibodies that potently inhibit parasite growth and impede interaction between PfRipr and its erythrocyte receptor. This is of considerable importance since the region was produced in a scalable and good manufacturing practice (GMP) compatible baculovirus-based vaccine production system allowing further vaccine development targeted adjustments. As a short and less complex protein that can be easily synthesized, PfRipr_5 is a potential candidate for further development as a blood-stage malaria vaccine.

## Materials and methods

### Production of recombinant proteins and antisera

An Ecto-PfRipr (Pf3D7_0323400; D_21_-N_1086_) gene with a C-terminal His-tag was synthesized as a wheat-codon optimized nucleotide sequence by GenScript (Tokyo, Japan) (Table S1). PfRipr truncates (Regions 1–11; Fig. 2a and Table S2) were amplified from the wheat codon optimized Ecto-PfRipr DNA sequence. All primer sequences used in this study are summarized in Table S2. The amplified DNA fragments with C-terminal His-tag sequence were restricted by XhoI and NotI, then ligated into a WGCFS plasmid (pEU-E01-GST-TEV-N2; CellFree Sciences, Matsuyama, Japan) for expression of GST-fusion proteins. The proteins were expressed with WGCFS (CellFree Sciences) as N-terminal glutathione S-transferase (GST) fusion proteins with C-terminal His-tags as described^49^. Ecto-PfRipr and PfRipr truncate recombinant proteins were purified using a Ni^2+^ Sepharose affinity column (GE Healthcare) as described^50^.

For GIA studies, Ecto-PfRipr was also expressed with WGCFS after ligation of the synthetic Ecto-PfRipr gene (Table S1) into the pEU-E01-MCS vector (CellFree Sciences, Matsuyama, Japan). For further experiments, PfRipr region 5 (PfRipr_5: C_720_ - D_934_) with His-tag at the C-terminus was synthesized as an insect-cell codon optimized gene (Table S1). The gene was cloned into the pFastBac1 expression vector and expressed using a baculovirus protein expression system (Genscript, Piscataway, NJ, USA). PfRipr_5 released into Sf9 cell culture supernatant was purified by a Ni-NTA column followed by a Superdex 200 column (GE Healthcare).

Parasite and erythrocyte surface proteins were expressed with WGCFS as N-terminal glutathione S-transferase (GST) fusion proteins with C-terminal His-tags. Specifically, the parasite proteins Rh5 (E_26_-Q_526_) and CyRPA (N_27_-E_362_), and the erythrocyte surface proteins AMIGO2, M_1_-N_397_; BCAM, E_32_-A_555_; CD44, Q_21_-_E606_; CD47, K_24_-S_139_; CD55, D_35_-T_381_; CD58, S_30_-R_215_; CD59, L_26_-P_128_; CD99, D_27_-G_125_; ERMAP, H_30_-S_154_; F11 receptor (F11R), H_32_-V_238_; ICAM4, A_23_-G_272_; NPTN, Q_29_-L_221_; and SEMA7A, Q_45_-H_666_. The recombinant proteins were purified using glutathione-Sepharose 4B columns (GE Healthcare) as described^50^.

GST-fused Rh5 proteins were prepared for Biocore single kinetics analysis by eluting GST-fused Rh5 bound to a glutathione-Sepharose 4B column using tobacco etch virus (TEV) protease (Invitrogen), which cleaves the TEV recognition site located between the GST tag and the Rh5 fragment, thereby releasing Rh5 from the N-terminal GST tag.

To generate antisera, each affinity purified recombinant protein or truncate was used to immunize animals. All rat immunizations were conducted at Sumitomo Dainippon Pharma Co., Ltd. (Osaka, Japan); specifically, with Ecto-PfRipr (D_21_-N_1086_) and PfRipr truncates 1 (PfRipr_1) amino acid (aa), D_21_-A_197_; 2, I_198_-K_377_; 3, K_378_-N_557_; 4, M_558_-K_719_; 5, C_720_-D_934_; 6, G_935_-N_1086_; 7, T_108_-R_287_; 8, C_288_-I_467_; 9, F_468_-N_647_; 10, S_648_-C_830_; and 11, S_831_-I_1007_ (based on the 3D7 isolate gene sequence; Pf3D7_0323400). Six Sprague-Dawley rats (Charles River Laboratories Japan, Inc.) per group were immunized sub-cutaneously with each antigen (10 µg) emulsified in Freund’s complete adjuvant, followed by booster immunizations in Freund’s incomplete adjuvant at three-week intervals. Antisera were collected three weeks after the last immunization. Rabbit anti-PfRipr_5 antiserum was purchased from Kitayama labes. Specifically, New Zealand White rabbits were immunized twice with antigen in Freund’s complete adjuvant at four-week intervals. Antisera were collected two weeks after the last immunization (Kitayama labes Co. Ltd. Ina, Japan).

Rabbit anti-PfRipr antibodies were purified by affinity chromatography by applying rabbit sera against PfRipr K_279_ -D_995_ to immobilized-recombinant PfRipr_5 coupled to a HiTrap NHS activated HP column (GE Healthcare) following the manufacturer’s instructions. All fractions were assayed for protein concentration and used in GIA.

### *P. falciparum* culture and transfection

Asexual stage *P. falciparum* 3D7 strain was a kind gift from the National Institute of Allergy and Infectious Diseases (NIAID), and was cultured as described^51^. To generate a parasite line expressing PfRipr-HA, full length *pfripr* was amplified by PCR with the primers indicated in Table S2 (Ripr-tran F and R). The DNA fragments were digested with SpeI and PstI, and ligated into pD3HA plasmids with a Pfef1-alpha promoter for episomal expression^52,53^. For transfection, parasitized erythrocytes were resuspended in 200 µl of cytomix (120 mM KCl, 0.15 mM CaCl_2_, 2 mM EGTA, 5 mM MgCl_2_, 10 mM K_2_HPO_4_/KH_2_PO_4_, 25 mM HEPES, pH 7.4) containing 100 µg of plasmid DNA. Electroporations were performed in 2 mm cuvettes using a Gene Pulser Xcell Electroporation System (Bio-Rad, Hercules, CA) at the condition 0.31 kV, 950 µF, and ∞ Ω. After electroporation the erythrocytes were immediately resuspended in complete medium. WR99210 was added to 10 nM in the culture medium one day post electroporation, and the culture maintained under the same drug pressure until drug-resistant parasites appeared.

### Preparation of parasite schizont extract and Western blot analysis

Western blot with schizont-rich parasite pellets from *P. falciparum* 3D7 strain was conducted as described^50^. Additional details are included in the Supplementary Methods.

### Imaging by indirect immunofluorescence assay (IFA)

IFA was conducted as described^50^ with rabbit anti-HA antibody (Abcam) at 1:100; mouse anti-AMA1 antibody at 1:100; and secondary antibodies, Alexa Fluor 488-conjugated goat anti-rabbit IgG and Alexa Fluor 568-conjugated goat anti-mouse IgG (Invitrogen), at 1:1000.

### Erythrocyte binding assay (EBA)

Erythrocyte binding assays were performed with native EBA175 and PfRipr shed into the culture supernatant of transfected parasites as described^40,54^. Additional details are included in the Supplementary Methods. Fresh human erythrocytes were washed three times in incomplete RPMI medium (iRPMI; RPMI 1640 medium with L-glutamine, 25 mM HEPES buffer, and 50 mg/l of hypoxanthine without sodium bicarbonate; Invitrogen) before enzyme treatment. Subsequently, 90 µl of concentrated (×20) culture supernatant was incubated with 10 µl of untreated and enzyme-treated human erythrocytes on a rotating wheel for 60 min at RT. After incubation the tube was centrifuged at 2,000 × g for 5 min at 4 °C and the supernatant was removed. The pellet was incubated with 200 µl of tetanolysin solution (final concentration of 1 µg/ml in iRPMI) for 10 min at 37 °C for hemolysis, and the reaction mixture was centrifuged at 13,000 × g for 10 min to collect erythrocyte membranes. After repeating the centrifugation three times, the erythrocyte membranes were resuspended in 200 µl of iRPMI and transferred to a new tube. The tube was centrifuged at 13,000 × g for 10 min and 60 µl of reducing SDS-PAGE sample buffer added after the removal of the supernatant. The samples were incubated at 37 °C for 30 min and subsequently resolved by SDS-PAGE. Following Western blotting as described above, native PfRipr and EBA175 proteins were detected by the respective rabbit antibodies.

For the EBA with recombinant proteins (Ecto-PfRipr and His-GST), each recombinant protein was incubated in 100 µl of untreated and enzyme-treated human erythrocytes with 50% hematocrit (final concentration of recombinant protein was 3.5 pM). Protein bound to erythrocytes was determined as described above for the EBA with native protein.

### Growth inhibition assay (GIA)

The inhibitory activity of the total IgGs from rat antisera against the recombinant proteins on merozoite invasion was determined over one cycle of 3D7 parasite replication. Parasitemia was determined by flow cytometry^50^. Rat antibody to anti-His-GST was used as a negative control. GIA experiments with N-terminal GST-fused recombinant PfRipr protein were conducted similarly at a final protein concentration of 5 µM. His-GST at the same concentration was used as the negative control. For each assay, three independent experiments were carried out in triplicate to confirm reproducibility.

### Protein-protein interactions by AlphaScreen

Interaction between PfRipr and 13 erythrocyte surface proteins was quantified by AlphaScreen as reported^40^. Briefly, reactions were carried out in 20 µl of reaction volume per well in 384-well OptiPlate microtiter plates (PerkinElmer). First, affinity-purified Ecto-PfRipr recombinant protein was biotinylated using a Biotin Labeling Kit-NH_2_ (Dojindo Molecular Technologies, Kumamoto, Japan) according to the manufacturer’s instruction. Secondly, 5 µl of 10 nM biotinylated protein was mixed with 5 µl of 10 nM for each erythrocyte surface protein in reaction buffer (100 mM Tris-HCL [pH 8.0], 0.01% [v/v] Tween-20 and 0.1 mg/ml [w/v] bovine serum albumin), and incubated for 1 h at 26 °C to form a protein-protein complex. Subsequently, a 10 µl suspension of streptavidin-coated donor-beads and anti-GST acceptor-beads (PerkinElmer) mixture in 1:1 (v/v) in the reaction buffer was added to a final concentration of 15 µg/ml of both beads. The mixture was incubated at 26 °C for 12 h in the dark to allow the donor- and acceptor-beads to optimally bind to biotin and GST, respectively. Upon illumination of this complex, a luminescence signal at 620 nm was detected by the EnVision plate reader (PerkinElmer) and the results were expressed as AlphaScreen counts. GST tagged Rh5, known to interact with PfRipr, was included as a positive control and His-GST as a negative control.

### Surface plasmon resonance (SPR)

All SPR experiments were performed using a Biacore X100 instrument (GE Healthcare) according to the manufacturer’s instructions and as reported^40^. Additional details are included in the Supplementary Methods.

### Immunoprecipitation

Erythrocyte ghosts were prepared as reported^55^. Recombinant proteins (either GST-PfRipr or GST-AMA1, 3 μM, 100 μl) and erythrocyte ghosts (100 μg) were incubated in lysis buffer (50 mM Tris-HCl, 0.2 M NaCl, 5 mM EDTA, 0.5% NP-40, cOmplete^TM^ protease inhibitor) at 37 °C for 1 hour. Protein G conjugated magnetic beads (25 µl; Thermo Fisher Scientific, San Jose, CA) were then added and further incubated for 30 min to remove nonspecific binding. The beads were precipitated with a magnetic apparatus and the supernatant sample (200 µl) transferred to a new tube containing 25 µl of magnetic beads preincubated with 5 µl of rabbit anti-SEMA7A polyclonal antibody (Abcam, Cambridge, UK). The mixture was incubated at 37 °C for 30 min. The beads were washed three times with 500 µl of wash buffer (50 mM Tris-HCl, 0.2 M NaCl, 5 mM EDTA, 0.5% NP-40). Finally, proteins were extracted from the protein G-conjugated beads by incubation with 50 µl of 1× SDS-PAGE reducing loading buffer at 37 °C for 30 min. Final supernatant was used for Western blot analysis. Similarly, PfRipr-HA was immunoprecipitated using rabbit anti-HA antibody (Abcam) from schizont-rich parasite lysates.

### Ethical approval

Human erythrocytes and plasma were procured from the Japanese Red Cross Society, and their use for parasite culture and *in vitro* experiments approved by the Ethical Review Committee of Ehime University Hospital (Aidaiibyourin 1301005). Animal experiments at Sumitomo Dainippon Pharma Co., Ltd were approved by Sumitomo Dainippon Pharma Ethical Review Committee (approval Number AN12314). All experiments were conducted in accordance with approved protocols and regulations.

## Supplementary information

Supplementary Methods, Tables and Figures.

## Data Availability

The datasets generated and analyzed during the current study are available from the corresponding author on reasonable request.
