# Supplementary material for: Antibodies against a short region of PfRipr inhibit Plasmodium falciparum merozoite invasion and PfRipr interaction with Rh5 and SEMA7A
Source: Sci Rep. 2020 Apr 20;10:6573. doi: 10.1038/s41598-020-63611-6 (PMC7171142; doi:10.1038/s41598-020-63611-6)
Supplement: Supplementary file 1 — Supplementary Methods, Tables and Figures. [file 41598_2020_63611_MOESM1_ESM.pdf]

Supplementary Materials and Methods, Tables, and Figures

Antibodies against a short region of PfRipr inhibit *Plasmodium falciparum* merozoite invasion and PfRipr interaction with Rh5 and SEMA7A

Hikaru Nagaoka[1], Bernard N. Kanoi[1], Edward H. Ntege[1#a], Masamitsu Aoki[2], Akihisa Fukushima[2], Takafumi Tsuboi[1], Eizo Takashima[1*]

[1]Division of Malaria Research, Proteo-Science Center, Ehime University, 3 Bunkyo-cho, Matsuyama, Japan,

[2]Sumitomo Dainippon Pharma Co., Ltd, 3-1-98, Kasugadenaka, Konohanaku, Osaka 554-0022, Japan.

[#a] Current Address: Department of Plastic and Reconstructive Surgery, University of the Ryukyus School of Medicine and Hospital, Okinawa, Japan

*Corresponding author: Eizo Takashima

Address: Division of Malaria Research, Proteo-Science Center, Ehime University, Japan, 3 Bunkyo-cho, Matsuyama, 790-8577, Japan.

Tel.: +81-89-927-9939

E-mail: eizo.takashima.mz@ehime-u.ac.jp

Supplementary materials and methods

Preparation of parasite schizont extracts and Western blot analysis

Western blot analyses were conducted as described [1]. Briefly, purified schizont-rich parasite pellets from the *P. falciparum* 3D7 strain were directly lysed in reducing SDS-PAGE sample buffer. The lysate was incubated at 37°C for 30 min, centrifuged at 10,000 × g for 10 min at 4˚C, supernatant collected, and resolved by SDS-PAGE on a Bolt™ 4-12 % Bis-Tris Plus (Invitorogen). Following electroblotting onto a polyvinylidene difluoride membrane with 0.2 μm pore size (Hybond LFP; GE Healthcare) with a semidry blotting system (Bio-Rad, Hercules, CA), the membrane was incubated with primary antibodies diluted at 1:1000 antisera to wash buffer ratio of rabbit anti-HA antibody (Abcam), rabbit anti-EBA175 (region III-V) antibody, rat anti-PfRipr antibody, rabbit anti-GST antibody (Santa Cruz Biotechnology), and rat antibodies to each of the PfRipr truncates. After washing, the membrane was probed with horseradish peroxidase-conjugated secondary-antibody (GE Healthcare) and visualized with Immobilon Western chemiluminescent horseradish peroxidase substrate (Millipore, Billerica, MA) on a LAS 4000 Mini luminescent-image analyzer (GE Healthcare). The relative molecular masses of the proteins were estimated with reference to Precision Plus protein standards (Bio-Rad). Where stated, striped membrane was washed and blocked before re-probing with a second antibody.

Erythrocyte binding assay (EBA)

Human erythrocytes were washed three times in incomplete RPMI medium (iRPMI; RPMI 1640 medium with L-glutamine, 25 mM HEPES buffer, and 50 mg/l of hypoxanthine without sodium bicarbonate (Invitrogen)) before use. Enzyme treatments of erythrocytes were done as described [1].

Erythrocyte binding assays (EBA) were performed with native EBA175 and PfRipr shed in the culture supernatant of transfected parasites. Ninety μl of concentrated (×20) culture supernatant was incubated with 10 μl of untreated and enzyme-treated human erythrocytes on a rotating wheel for 60 min at RT. After incubation the tube was centrifuged at 2,000 × g for 5 min at 4˚C and supernatant was removed. The pellet was incubated with 200 μl of tetanolysin solution (final concentration of 1 μg/ml in iRPMI) for 10 min at 37°C for hemolysis, and the reaction mixture was centrifuged at 13,000 × g for 10 min to collect erythrocyte membranes. After repeating the centrifugation a total of three times, the erythrocyte membrane was resuspended in 200 μl of iRPMI and transferred to a new tube. The tube was centrifuged at 13,000 ×g for 10 min and 60 μl of reducing SDS-PAGE sample buffer added after the removal of the supernatant. The samples were incubated at 37°C for 30 min and subsequently resolved by SDS-PAGE. Following Western blotting as described above, native PfRipr and EBA175 proteins were detected by the respective rabbit antibodies as primary antibodies.

For EBA with recombinant proteins (Ecto-PfRipr and His-GST), each recombinant protein was incubated in 100 μl of untreated and enzyme-treated human erythrocytes with 50% hematocrit (final concentration of recombinant protein was 3.5 pM). The abundance of protein bound to the erythrocytes was determined as described for EBA with native proteins above.

Protein-protein interactions by AlphaScreen

Interaction between PfRipr and the 13 erythrocyte surface proteins was quantified by AlphaScreen as described [2]. Briefly, reactions were carried out in a 20 μl reaction volume per well in 384-well OptiPlate microtiter plates (PerkinElmer). First, affinity-purified Ecto-PfRipr recombinant protein was biotinylated using a Biotin Labeling Kit-NH_2 (Dojindo Molecular Technologies, Kumamoto, Japan) according to the manufacturer's instruction. Secondly, 5 μl of 10 nM biotinylated protein was mixed with 5 μl of 10 nM each erythrocyte surface protein

in reaction buffer (100 mM Tris-HCL [pH 8.0], 0.01% [v/v] Tween-20 and 0.1 mg/ml [w/v] bovine serum albumin), and incubated for 1 h at 26°C to form a protein-protein complex. Subsequently, a 10 μl suspension of streptavidin-coated donor-beads and anti-GST acceptor-beads (PerkinElmer) mixture in 1:1 (v/v) in the reaction buffer was added to a final concentration of 15 μg/ml of both beads. The mixture was incubated at 26°C for 12 h in the dark to allow the donor- and acceptor-beads to optimally bind to biotin and GST, respectively. Upon illumination of this complex, a luminescence signal at 620 nm was detected using an EnVision plate reader (PerkinElmer) and the results were expressed as AlphaScreen counts. GST tagged Rh5, known to interact with PfRipr, was included as a positive control while His-GST as a negative control.

Surface plasmon resonance (SPR)

All SPR experiments were performed using a Biacore X100 instrument (GE Healthcare) according to the manufacturer's instructions and as reported (ref 56). The Biacore X100 evaluation software was used for single-cycle or affinity binding analysis. Sensor CM5, amine coupling reagents, GST capture kit, and buffers were purchased from GE Healthcare. Fresh HBS-EP+ (10 mM HEPES, pH 7.4, 150 mM NaCl, 3 mM EDTA, 0.05% (v/v) surfactant P20) was used as a running buffer for all SPR experiments. Blank flow cells were used to subtract buffer effects on sensorgrams. After subtraction of the contribution of bulk refractive index, and nonspecific interactions with the CM5 chip surface, individual association (k_a) and dissociation (k_d) rate constants were obtained by global fitting of data. Measurement conditions were optimized so that the contribution of mass transport to the observed values of K_D was negligible.

Recombinant Ecto-PfRipr immobilized on a sensor CM5 chip was used as the ligand while either Rh5, CyRPA, His-GST, SEMA7A, AMIGO2, or NPTN was used as analyte. All

assays were performed at increasing analyte concentrations of 3.13, 6.25, 12.5, 25, and 50 nM at a contact time of 120 s and dissociation time of 180 s with the last dissociation extended up to 3000 s.

The potency of anti-PfRipr_5 antibodies to inhibit interaction between recombinant Ecto-PfRipr and either recombinant Rh5, CyRPA, or SEMA7A, was also assessed, Specifically, recombinant GST-fused Rh5, CyRPA, or SEMA7A proteins at 300 nM were captured individually on SPR sensor chips using a GST-capture kit, Ecto-PfRipr protein was added as followed by addition of analyte consisting of 300 nM of recombinant and 300 nM of anti-PfRipr_5 antibodies.

1 Arumugam, T. U. *et al.* Discovery of GAMA, a *Plasmodium falciparum* merozoite micronemal protein, as a novel blood-stage vaccine candidate antigen. *Infect Immun* **79**, 4523-4532, doi:10.1128/IAI.05412-11 (2011).

2 Nagaoka, H. *et al.* PfMSA180 is a novel *Plasmodium falciparum* vaccine antigen that interacts with human erythrocyte integrin associated protein (CD47). *Sci Rep* **9**, 5923, doi:10.1038/s41598-019-42366-9 (2019).

Table S1: PfRipr Codon optimized sequence for expression with Wheat Germ Cell Free System

```
ATGGATCTGATCGAGGGCATCTTCTACGAGAAGAACGAGATCGACAAGCTCACCTTCTCCCTGGACCACAGGGTCCGCGATAACCTCAAGACG
GACCTCATCCTGAACAACAACGGCGAGAACGATTACGCGTACCTGAACAAGTATGTGTACACCATCCTCAACAGGGACTCAACCGAGAAGATC
AAGACGTTCTTCTCTCACAACAAGGATATGAAGTCTTGCGACTACTTCATCTCCAAGGAGTACAACTCCAGCGATAAGACCAACCAAATCTGC
TACAAGAAGACGTTCTGCGGCGTGGTCATCCCAAACTCCGAGGAGATCAAGACCAACAAGATCACGAACGACAAGCTCTACTGCGCCCATTTC
AACTCCACCCACATCATCATCTACTACATCAGCCAGCCACTCCTGCTGGAGCCGCACGTTGTGTACGAGGAGACCTTCTTCGAGAAGGGCAAG
AACGACCAAATCAACTGCCAGGGGATGTACATCTCACTGCGGTCTGTCCACGTTCATACCCACAACGCGATCCTCCAACAGGAGACCCTGACG
TACATCAAGAACCTCTGCGACGGCAAGAACAACTGCAAGTTCGACTTCGATAGCATCAAGTACGAGAACAAGTCGCTCACCCACTACCTGTTC
TTCATCAACATCCAGTACCAGTGCATCTCCCCGCTCAACCTGCAAGAGAACGAGATGTGCGACGTCTACAACGACGATACCCACAAGGCCACG
TGCAAGTACGGCTTCAACAAGATCGAGCTGCTCAAGAACGTCTGCGAGGAGAACTACCGCTGCACCCAAGATATCTGCTCCGTTAACCAGTTC
TGCGACGGCGAGAACGAGACCTGCACGTGCAAGACGTCGCTGCTCCCCTCAGCGAAGAACAACTGCGAGTACAACGACCTCTGCACCGTGCTG
AACTGCCCTGAGAACAGCACGTGCGAGCAGATCGGCAACGGGAAGAAGGCTGAGTGCAAGTGCGAGAACGGCAAGTACTACCATAACAACAAG
TGCTACACCAAGAACGACCTGGAGCTGGCGATCAAGATCGAGCCACACAAGAAGGAGAAGTTCTACAAGAACAACCTCTACCAAGGCAAGGCT
CTGAAGCCGGAGTACATCTTCATGCAGTGCGAGAACGGGTTCAGCATCGAGGTCATCAACGCCTACGTCTCGTGCTACCGCGTTTCATTCAAC
CTCAACAAGCTGAAGTATGTGACCGAGTCCCTGAAGAAGATGTGCGATGGCAAGACCAAGTGCGCGTACGGGAACACGATCGACCCAATCGAC
GATCTCAACCACCATAACATCTGCAACAACTTCAACACCATCTTCAAGTACGATTACCTGTGCGTGTTCAACAACCAAAACATCACGTCTGAC
AAGAACTCCCATCTCCACAGCAACATCCCCTCGCTCTACAACTCGTCAATCCTGCCTGACATCAACAAGTCGAAGTTCCATCTGATCTCCAGG
AACAGCCGCACCAACCAGTACCCACACAACAACATCTCCATGCTGGAGATCCAAAACGAGATCTCTTCCCATAACTCAAACCAGTTCTCTACC
GACCCGCACACGAACAGCAACAACATCAACAACATGAACATCAAGAAGGTCGAGATCTTCCGGTCGAGATTCAGCTCGAAGCTGCAATGCCAG
GGCGGGAAGATCAACATCGATAAGGCTATCCTCAAGGGCGGGGAGGGCTGCAACGACCTGCTCCTGACCAACTCGCTCAAGTCATACTGCAAC
GATCTGAGCGAGTGCGACATCGGGCTCATCTACCATTTCGATACCTACTGCATCAACGACCAATACCTGTTCGTGTCCTACAGCTGCTCGAAC
CTCTGCAACAAGTGCCACAACAACTCCACCTGCTACGGCAACAGGTTCAACTACGACTGCTTCTGCGATAACCCATACATCAGCAAGTACGGG
AACAAGCTGTGCGAGCGCCCGAACGATTGCGAGTCCGTGCTCTGCAGCCAAAACCAGGTCTGCCAAATCCTCCCAAACGACAAGCTGATCTGC
CAGTGCGAGGAAGGCTACAAGAACGTGAAGGGGAAGTGCGTCCCAGACAACAAGTGCGATCTGTCTTGCCCGTCCAACAAGGTGTGCGTCATC
GAGAACGGCAAGCAGACCTGCAAGTGCAGCGAGCGGTTCGTTCTGGAGAACGGCGTGTGCATCTGCGCTAACGATTACAAGATGGAGGACGGG
ATCAACTGCATCGCCAAGAACAAGTGCAAGAGAAAGGAGTACGAGAACATCTGCACCAACCCCAACGAGATGTGCGCTTACAACGAGGAGACG
GACATCGTCAAGTGCGAGTGCAAGGAGCACTACTACCGGTCATCTAGAGGCGAGTGCATCCTCAACGACTACTGCAAGGATATCAACTGCAAG
GAGAACGAGGAGTGCTCCATCGTTAACTTCAAGCCTGAGTGCGTGTGCAAGGAGAACCTGAAGAAGAACAACAAGGGCGAGTGCATCTACGAG
AACTCTTGCCTCATCAACGAGGGGAACTGCCCCAAGGACTCCAAGTGCATCTACAGGGAGTACAAGCCTCATGAGTGCGTGTGCAACAAGCAA
GGCCACGTTGCCGTGAACGGGAAGTGCGTCCTGGAGGACAAGTGCGTTCACAACAAGAAGTGCAGCGAGAACTCGATCTGCGTCAACGTTATG
AACAAGGAGCCCATCTGCGTGTGCACCTACAACTACTACAAGAAGGATGGCGTCTGCCTCATCCAGAACCCTTGCCTGAAGGACAACGGCGGG
TGCTCACGCAACTCTGAGTGCACCTTCAAGTACTCAAAGATCAACTGCACGTGCAAGGAGAACTACAAGAACAAGGACGATTCTTGCGTGCCC
AACACCAACGAGTACGACGAGTCGTTCACGTTCCAATACAACGACGATGCCTCAATCATCCTCGGCGCGTGCGGGATGATCGAGTTCTCCTAC
ATCTACAACCAGATCATCTGGAAGATCAACAACTCCAAGGAGAGCTACGTTTTCTACTACGACTACCCGACCGCCGGCAACATCGAGGTGCAA
ATCAAGAACGAGATCTTCCATACCATCATCTACCTCAAGAAGAAGATCGGGAACTCAGTCATCTACGACGATTTCCAAGTTGACCACCAGACC
TGCATCTACGAGAACGTGTTCTACTACTCCAACCAGAACCACCATCACCATCACCATTGA
```

Codon optimized PfRipr_5 sequence for expression with Baculovirus protein expression system

```
ATGCTCCTCGTCAACCAGTCACATCAAGGCTTCAACAAAGAACATACCTCGAAAATGGTCTCCGCAATCGTGCTCTACGTGCTGCTCGCTGCC
GCAGCGCACTCAGCTTTCGCCTGCGACTTGTCTTGTCCCTCAAACAAGGTGTGCGTCATCGAGAACGGAAAGCAGACCTGCAAATGTTCCGAG
AGGTTCGTGCTGGAAAACGGAGTCTGCATTTGTGCAAACGACTACAAGATGGAGGATGGTATCAACTGCATTGCCAAGAACAAGTGTAAGAGA
AAAGAGTACGAAAACATCTGCACCAACCCAAACGAAATGTGCGCTTACAACGAGGAAACTGATATTGTGAAGTGCGAGTGTAAAGAACATTAC
TACCGCTCCAGCCGTGGCGAGTGCATCCTCAACGACTACTGCAAGGATATTAACTGTAAAGAAAACGAGGAATGCTCTATCGTCAACTTCAAG
CCGGAGTGCGTTTGTAAGGAAAACCTGAAGAAAAACAACAAGGGCGAGTGCATCTACGAAAACTCCTGTCTCATTAACGAGGGAAACTGCCCT
AAGGACAGCAAATGTATCTACCGCGAGTACAAGCCCCACGAATGCGTCTGTAACAAGCAAGGTCATGTTGCTGTGAACGGCAAGTGCGTTCTG
GAGGATAAATGTGTGCACAACAAGAAATGCAGTGAAAACTCGATCTGTGTCAACGTTATGAACAAGGAGCCCATCTGTGTCTGCACCTACAAC
TACTACAAAAAGGACCATCATCATCATCATCAC
```

Table S2: List of PCR primers used in this study.

for pD	Primer sequence	Insert (amino acids)
Ripr-tran F	gagagagaACTAGTATGTTCAGAATTTTTTTTACCCTTCTTATAATAATATTAATCAAG	
Ripr-tran R	gagagagaCTGCAGCATTCTGATTACTATAATAAAATACATTTTCATATATACATGTTTGATG	M1 - N1086
for pEU		
CyRPA F	gagagagaCTCGAGATAAATTGTGATAGTCGTCATGTTTTTATAAGGA	
CyRPA R	gagagagaGCGGCCGCTCAATGGTGATGGTGATGGTGCTCATAGTTAGCATAGTATATAGTATGAATATTATATATCCCTTG	I26 – E362
PfRipr_1 F	gagagagaCTCGAGATGGATCTGATCGAG	
PfRipr_1 R	gagagagaGCGGCCGCTCAATGGTGATGGTGATGGTGCGCGTTGTGGGTATGAAC	D21 – A197
PfRipr_2 F	gagagagaCTCGAGATGATCCTCCAACAGGAGACC	
PfRipr_2 R	gagagagaGCGGCCGCTCAATGGTGATGGTGATGGTGCTTGTGTGGCTCGATCTTG	I198 – K377
PfRipr_3 F	gagagagaCTCGAGATGAAGGAGAAGTTCTACAAGAACAAC	
PfRipr_3 R	gagagagaGCGGCCGCTCAATGGTGATGGTGATGGTGGTTGTTGATGTTGTTGCTGTTC	K378 – N557
PfRipr_4 F	gagagagaCTCGAGATGAACATCAAGAAGGTCGAG	
PfRipr_4 R	gagagagaGCGGCCGCTCAATGGTGATGGTGATGGTGCTTGTTGTCTGGGACGC	M558 – K719
PfRipr_5 F	gagagagaCTCGAGATGTGCGATCTGTCTTGCCC	
PfRipr_5 R	gagagagaGCGGCCGCTCAATGGTGATGGTGATGGTGATCCTTCTTGTAGTAGTTGTAGGTG	C720 – D934
PfRipr_6 F	gagagagaCTCGAGATGGGCGTCTGCCTCATCC	
PfRipr_6 R	gagagagaGCGGCCGCTCAATGGTGATGGTGATGGTGGTTCTGGTTGGAGTAGTAGAACAC	G935 – N1086
PfRipr_7 F	gagagagaCTCGAGATGACCAACCAAATCTGCTACAAG	
PfRipr_7 R	gagagagaGCGGCCGCTCAATGGTGATGGTGATGGTGGCGGTAGTTCTCCTCGC	T108 – R287
PfRipr_8 F	gagagagaCTCGAGATGTGCACCCAAGATATCTGCTC	
PfRipr_8 R	gagagagaGCGGCCGCTCAATGGTGATGGTGATGGTGGATGGTGTTGAAGTTGTTGCAG	C288 – I467
PfRipr_9 F	gagagagaCTCGAGATGTTCAAGTACGATTACCTGTGC	
PfRipr_9 R	gagagagaGCGGCCGCTCAATGGTGATGGTGATGGTGGTTGTTGTGGCACTTGTTG	F468 – N647
PfRipr_10 F	gagagagaCTCGAGATGTCCACCTGCTACGGC	
PfRipr_10 R	gagagagaGCGGCCGCTCAATGGTGATGGTGATGGTGGCACTCCTCGTTCTCCTTG	S648 – C830
PfRipr_11 F	gagagagaCTCGAGATGTCCATCGTTAACTTCAAGCCTG	
PfRipr_11 R	gagagagaGCGGCCGCTCAATGGTGATGGTGATGGTGGATCATCCCGCACGCG	S831 – I1007
AMIGO2_F	GGGCGGATATCTCGAATGTCGTTACGTGTACACAC	
AMIGO2_R	AAAACTAGTGCGGCCCTAATGGTGATGGTGATGGTGGTTAAATGCCTCATGAGCAT	M1 - N397
BCAM_F	GGGCGGATATCTCGAGAGGTGCGCTTGTCTGTA	
BCAM_R	AAAACTAGTGCGGCCCTAATGGTGATGGTGATGGTGGCCACGGCCATGACGGCC	E32 – A555
CD44_F	GGGCGGATATCTCGACAGATCGATTTGAATATAACC	
CD44_R	AAAACTAGTGCGGCCCTAATGGTGATGGTGATGGTGTTCTGGAATTTGGGGTGTC	Q21 – E606
CD47_F	GGGCGGATATCTCGAAAAACAAAATCTGTAGAATTCAC	
CD47_R	AAAACTAGTGCGGCCCTAATGGTGATGGTGATGGTGTGGAGAAAACCATGAAACAAC	K24 – S139
CD55_F	GGGCGGATATCTCGAGACTGTGGCCTTCCCCCA	
CD55_R	AAAACTAGTGCGGCCCTAATGGTGATGGTGATGGTGAGTCAGCAAGCCCATGGT	D35 – T381
CD58_F	GGGCGGATATCTCGATCCCAACAAATATATGGTGTT	
CD58_R	AAAACTAGTGCGGCCCTAATGGTGATGGTGATGGTGTCTGTGTCTTGAATGACCG	S30 – R215
CD59_F	GGGCGGATATCTCGACTGCAGTGCTACAACTGTC	
CD59_R	AAAACTAGTGCGGCCCTAATGGTGATGGTGATGGTGGGGATGAAGGCTCCAGGC	L26 – P128
CD99_F	GGGCGGATATCTCGAGATTTATCTGATGCCCTTCC	
CD99_R	AAAACTAGTGCGGCCCTAATGGTGATGGTGATGGTGGCCTGGGGCGTCGGCCTC	D27 – G125
ERMAP_F	GGGCGGATATCTCGACACGCAGGGGATGCCGGC	
ERMAP_R	AAAACTAGTGCGGCCCTAATGGTGATGGTGATGGTGTGAGGGGGAGAGACTCCC	H30 – S154
F11R_F	GGGCGGATATCTCGACACTCTTCTGAACCTGAAG	
F11R_R	AAAACTAGTGCGGCCCTAATGGTGATGGTGATGGTGGACCCCCACATTCCGCTC	H32 – V238
ICAM4_F	GGGCGGATATCTCGAGCGCTGGGACGCCGGACT	
ICAM4_R	AAAACTAGTGCGGCCCTAATGGTGATGGTGATGGTGGCCACCATGTATGGCCAT	A23 – G272
NPTN_F	GGGCGGATATCTCGACAGAACGAGCCAAGGATTG	
NPTN_R	AAAACTAGTGCGGCCCTAATGGTGATGGTGATGGTGCAGGTGGCTCCGCACCCT	Q29 – L221
SEMA7A_F	GGGCGGATATCTCGACAGGGCCACCTAAGGAGC	
SEMA7A_R	AAAACTAGTGCGGCCCTAATGGTGATGGTGATGGTGGTGGACCAGCAAGCCAAG	Q45 – H666

Figure S1

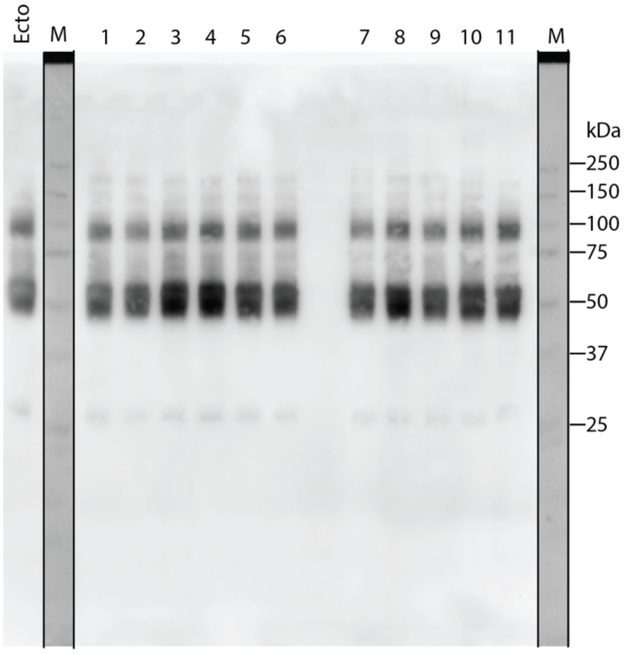

Figure S1

Western blot analysis of Native PfRipr. PfRipr expressed in the parasites was immuno-precipitated with rat antibodies to Ecto-PfRipr and each of the 11 PfRipr truncate regions. In the Western blot the immunoprecipitated PfRipr was detected with mouse anti-Ripr_5-BPES antibodies.

Figure S2

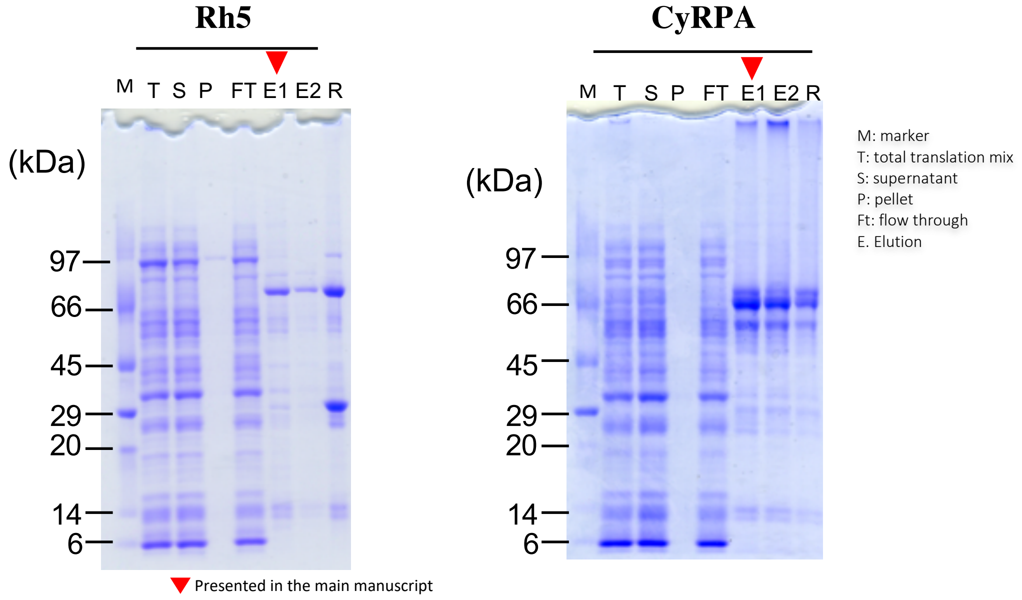

Figure S2

Recombinant Rh5 and CyRPA protein expression. Recombinant Rh5 and CyRPA proteins were expressed with WGCFS as GST-fused proteins with a C-terminal His-tag. The proteins were resolved by 12.5% SDS-PAGE under reducing condition and stained with CBB.

Figure S3

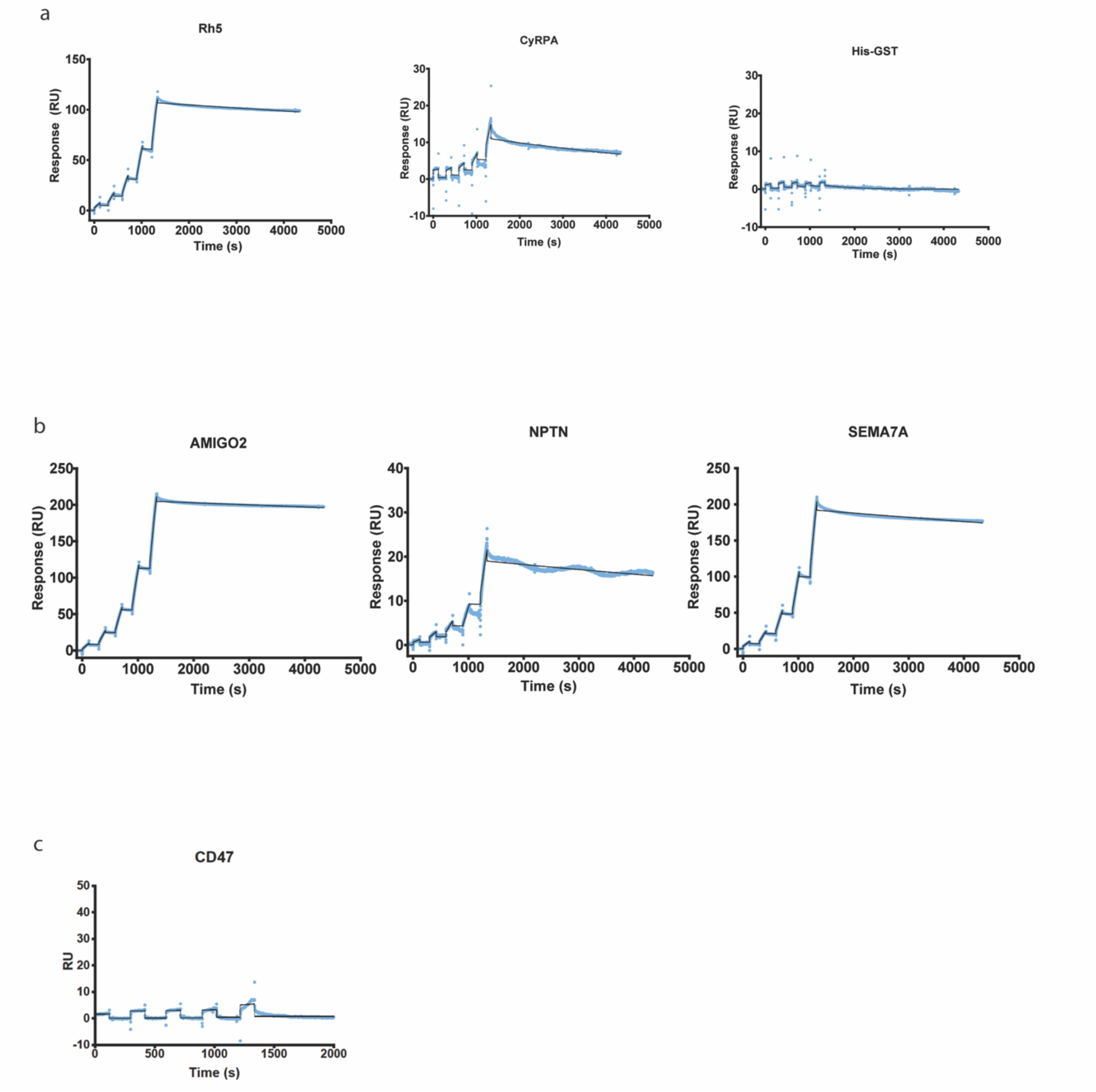

Figure S3

SPR single-cycle kinetic analysis sensorgrams. Recombinant Ecto-PfRipr immobilized on a sensor CM5 chip was used as the ligand while the indicated proteins were used as analyte. The blue curve represents the data-generated sensorgram while the black curve indicates line of fit used to calculate kinetics parameters. All assays were performed at increasing protein concentrations of 3.13, 6.25, 12.5, 25, and 50 nM at a contact time of 120 s and extended dissociation time of 3000 s.

Figure S4

a

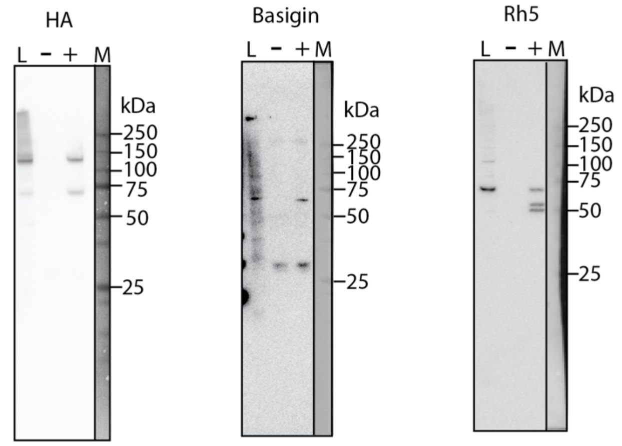

b

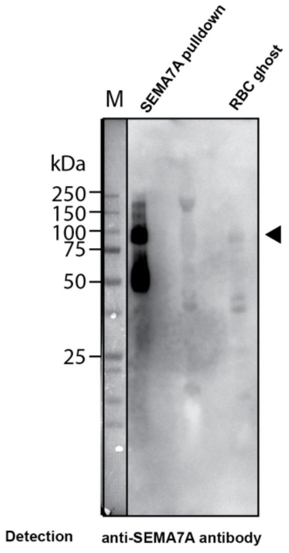

c

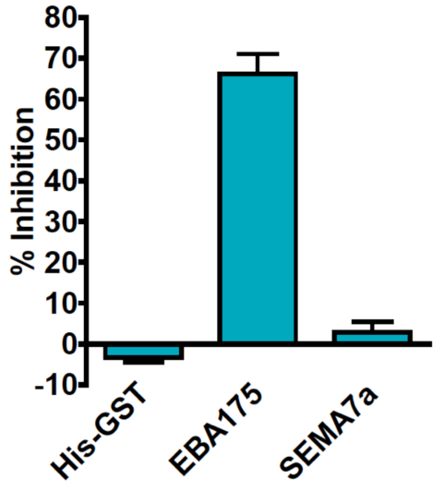

Figure S4

a. Western blot analysis: PfRipr-HA expressed in the parasites was immunoprecipitated with anti-HA antibody conjugated on the surface of magnetic beads. L, parasite lysate prepared from PfRipr-HA parasite; −, immunoprecipitated sample with 3D7 parasite; and +, immunoprecipitated sample with PfRipr-HA lysate. Western blotting was conducted with the indicated primary antibodies; namely, rat anti-HA antibody, mouse anti-basigin monoclonal antibody, and rabbit anti-Rh5 antibody. The same immunoprecipitation lysate per parasite line was used in the three membranes.

b. Western blot analysis: erythrocyte ghost sample and SEMA7A immunoprecipitated with rabbit anti-SEMA7A polyclonal antibodies were both probed with anti-SEMA7A polyclonal antibodies. Anti-SEMA7A antibody successfully pulled down SEMA7A. Arrowhead, SEMA7A.

c. GIA assay: Anti-SEMA7A antibody did not show GIA activity
